# The genomic and epidemiological investigations of enteric viruses of domestic caprine (*Capra hircus*) revealed the presence of multiple novel viruses related to known strains of humans and ruminant livestock species

**DOI:** 10.1128/spectrum.02533-23

**Published:** 2023-10-12

**Authors:** Ákos Boros, Péter Pankovics, Zoltán László, Péter Urbán, Róbert Herczeg, Gábor Gáspár, Fruzsina Tóth, Gábor Reuter

**Affiliations:** 1 Department of Medical Microbiology and Immunology, Medical School, University of Pécs, Pécs, Hungary; 2 János Szentágothai Research Centre of the University of Pécs, Bioinformatics Research Group, Genomics and Bioinformatics Core Facility, Pécs, Hungary; Changchun Veterinary Research Institute, Changchun, China

**Keywords:** picornavirus, astrovirus, IRES, livestock, CRESS DNA virus, epidemiology, ovine, caprine, cattle, NGS, phylogenetics, zoonosis

## Abstract

In this study, the enteric virome of diarrheic caprine was investigated using next-generation sequencing, reverse transcription PCR methods, and bioinformatics analyses. The complete or nearly complete genomes of seven novel viruses including (i) a caprine astrovirus (OQ758025) of family *Astroviridae*, four picornaviruses in genera (ii) *Boosepivirus* (OQ758026), (iii) *Enterovirus* (OQ758028), (iv) *Kobuvirus* (OQ758029), and (v) an unassigned picornavirus provisionally called as capripivirus (from the term caprine picornavirus) of the family *Picornaviridae* (OQ758027), as well as (vi) a tusavirus of family *Parvoviridae* (OL692339.2) and an (vii) unassigned CRESS DNA virus (OQ758030) have been identified. Structural analyses of the internal ribosomal entry sites revealed the presence of unique motifs and suggest the modular exchange of certain elements between co-infecting viruses. Epidemiological investigations and genotyping PCR reactions of identified RNA virus groups were also conducted using multiple “*in-house*” developed SYBRgreen-based screening quantitative PCR (qPCR) assays and generic capsid primers on additional fecal samples from ruminant livestock species (*n* = 62 caprine, *n* = 32 ovine, and *n* = 94 cattle) of three different age groups from *n* = 12 geographically distant sampling sites in Hungary. The results of qPCR screening assays as well as K-means cluster and phylogenetic analyses of the determined sequences suggest that diverse members of the above RNA virus groups were found in all age groups, but mostly in <1-year-old ruminants. Members of the analyzed virus groups were found frequently (37.2%) in multiple (even in quintuple) co-infections, while strains related closely to the identified index viruses were detectable only in certain caprine and ovine samples indicating a wide range of viral diversity.

**IMPORTANCE:**

Compared with other domestic animals, the virome and viral diversity of small ruminants especially in caprine are less studied even of its zoonotic potential. In this study, the enteric virome of caprine was investigated in detail using next-generation sequencing and reverse transcription PCR techniques. The complete or nearly complete genomes of seven novel viruses were determined which show a close phylogenetic relationship to known human and ruminant viruses. The high similarity between the identified caprine tusavirus (family *Parvoviridae*) and an unassigned CRESS DNA virus with closely related human strains could indicate the (reverse) zoonotic potential of these viruses. Others, like astroviruses (family *Astroviridae*), enteroviruses, or novel caripiviruses (named after the term caprine picornavirus) of family *Picornaviridae* found mostly in multiple co-infections in caprine and ovine, could indicate the cross-species transmission capabilities of these viruses between small ruminants.

## INTRODUCTION

Domestic caprine (*Capra aegagrus hircus*) together with ovine (*Ovis aries*) and cattle (*Bos taurus*) are related ruminant species of the family Bovidae. Although caprine and ovine belong to distinct genera and species of the subfamily Caprinae and possess different chromosome numbers (*Ovis* spp. have 54, while *Capra* spp. have 60 chromosomes) due to the close relationship, they are collectively referred to as small ruminants (1). Although there are a few known viruses of caprine, including some which have zoonotic potential like Orf virus or Rabies, compared to other livestock animals the virome of caprine is relatively less studied (1, 2).

The small circular, replication-associated protein (Rep) encoding single-stranded (ss) DNA viruses (CRESS DNA viruses) have remarkable genomic diversity and could infect various pro- and eukaryotic hosts including caprine, but only certain strains from the family *Circoviridae* and *Genomoviridae* as well as viruses from the “BMMF” (bovine meat and milk factors) circovirus group were described in goats (3 – 5). The 1–6-kb-long genomes of CRESS DNA viruses are encoding at least two proteins: a Rep and a capsid which are usually found in separate open reading frames (ORFs) of various orientations. The orthologous multifunctional Rep proteins with well-known amino acid motifs and a conserved origin of viral replication (*ori*) are characteristic features of eukaryotic CRESS DNA viruses (4, 6). The currently assigned CRESS DNA viruses belong to 12 different families of *Cressdnaviricota* phylum (https://ictv.global/taxonomy) although several recently discovered CRESS DNA viruses with unknown pathogenic and zoonotic potential from various hosts including humans are waiting for classification (6).

Parvoviruses (family *Parvoviridae*) are genetically highly diverse viruses which can infect animals and humans (7). The linear, 4–6-kb-long ssDNA genome contains a long coding region (for non-structural and capsid proteins) flanked by 120–600-nt terminal hairpins (7). Tusaviruses (genus *Protoparvovirus*) have been recently identified from a fecal sample of a diarrheic child in Tunisia and subsequently in enteric samples of diarrheic adults in Finland, but the exact pathogenic role of these viruses is unclear (8 – 10). Complete coding sequences (CDS) of caprine and ovine tusaviruses described recently (11) have up to 96% and 89% aa identity in NS1 and VP1, respectively, to human tusaviruses.

*Picornaviruses* of the family *Picornaviridae* are small non-enveloped viruses with a positive ssRNA genome. The c.a. 6.7–10.1-kb-long genome usually consists of a single ORF flanked by 5′ and 3′ UTRs and a 3′ terminal poly(A) tail (12). The picornaviruses contain a highly structured RNA element at the 5′UTR called an internal ribosomal entry site (IRES) which allows cap-independent translation of the viral ORF. Currently, five different IRES types (IRES I-V) could be distinguishable, each has a type-specific RNA structure and conserved nt motifs (13, 14). The viral polyprotein is predominantly organized in the following layout: (L)-**P1**:VP0 (or VP4-VP2)-VP3-VP1-**P2**:2A-2B-2C-**P3**:3A-3B-3C-3D (L: Leader protein, P1: capsid proteins, and P2-P3: non-structural proteins), although certain differences like the absence of a Leader protein (e.g., bopiviruses) or presence of multiple copies of 3B peptides (e.g., aphthoviruses) are also possible (12, 15, 16).

The family *Picornaviridae* is currently grouped into five sub-families with 68 genera (https://ictv.global/taxonomy, www.picornaviridae.com). Most of the caprine picornaviruses belong to 6 of the 68 total genera, including *Enterovirus* (species *Enterovirus F* and *G*), *Bopivirus* (species *Bopivirus B*), and *Kobuvirus* (species *Aichivirus C*) (16 – 22, www.picornaviridae.com) with mostly unknown pathogenic potential. There are some other genera like *Boosepivirus* which currently contains bovine (species *Boosepivirus A* and *B*) and ovine picornaviruses (*Boosepivirus C*) or genus *Erbovirus* whose members (Equine rhinitis B virus 1, 2, and 3) were identified only from horses but not from other species yet (23 – 25).

Astroviruses (AstVs) of the family *Astroviridae* are non-enveloped viruses with a +ssRNA genome. The AstVs have a general genome layout of 5′UTR-ORF1a/ORF1b-ORF2-3′UTR-poly(A) tail where the ORF1a and 1b are encoding the non-structural proteins 1a (NSP1a) and 1ab (NSP1ab) including the conserved RNA-dependent RNA polymerase (RdRp). The NSP1ab can be translated via a ribosomal skipping mechanism (26). The ORF2 encodes the capsid protein which is currently used for the *in silico* classification of AstVs into different genotype species [mean amino acid genetic distances (p-dist) between genotypes > 0.338] (27). To date, a total of 19 genotype species of genus *Mamastrovirus* (MAstV 1–19) have been officially recognized by the International Committee for Taxonomy of Viruses (ICTV) and a further 14 have been proposed (MAstV 20–33) but there are several other AstVs with still pending classification (26, 28). Astroviruses can be associated with gastroenteritis and disseminated infections in humans and ruminant species like cattle and ovine as well (26). The first caprine AstVs were described in 2019, but at present caprine, AstVs can be classified into six genotype species with unknown pathogenic role (29 – 31).

In this study, the eukaryotic enteric virome of three diarrheic caprine was investigated in detail using viral metagenomics and next-generation sequencing techniques together with different reverse transcription (RT-)PCR methods. Epidemiological investigations and PCR-based genotyping of the identified RNA viruses in additional enteric samples of ruminant species (caprine ovine and cattle) were also conducted using novel singleplex SYBRgreen (SYBRg)-quantitative PCR (qPCR) assays, generic capsid primer pairs, and various sequence analyses.

## RESULTS

### Overview of the next-generation sequencing data

**Fig 1:**
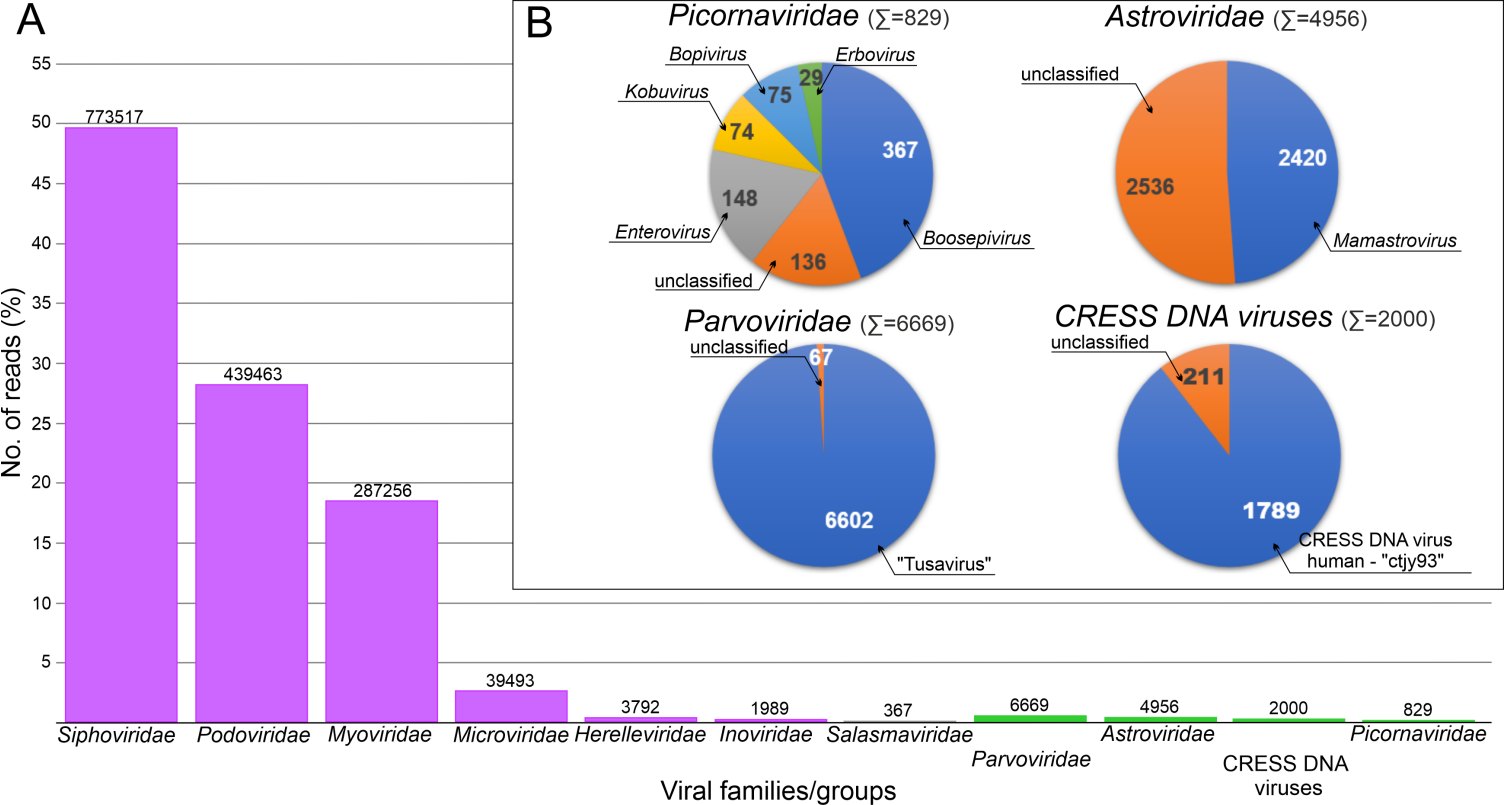
(**A**) Overview and classification of the viral reads acquired from the next-generation sequencing run (NovaSeq 6000, Illumina) of a pooled fecal specimen of three caprine (*Capra hircus*) with the sign of gastroenteritis. Violet and green bars indicate the abundance of identified potential bacterial (phages) and eukaryotic virus families/groups, respectively. Numbers above the bars indicate the number of reads that belong to a corresponding viral family/group. (**B**) Detailed classification of the reads of eukaryotic virus families/groups. Numbers in the pie charts indicate the number of reads most closely related to a corresponding genus or virus. “Unclassified” reads were unable to classify further to taxa below family level.

A total of 10,469,408 paired-end reads were acquired from the next-generation sequencing run of a pooled fecal specimen of three (sample IDs: KT-G4, KT-G5, and KT-FG3) caprine (*Capra hircus*) with the sign of gastroenteritis. From the acquired reads, a total of 1,560,331 sequences could be classified into any of the known virus families (Fig. 1A). A Total of 99.1% of the viral sequences (*n* = 1,545,877) most likely belongs to various phage families (Fig. 1A) and only 0.9% (*n* = 14,454) was classified as eukaryotic viral sequences. Most of them (*n* = 8,669) belonged to the ssDNA virus family of *Parvoviridae* (*n* = 6,669) and CRESS DNA viruses (*n* = 2,000) while the rest of the sequences belong to +ssRNA virus families of *Astroviridae* (*n* = 4,956) and *Picornaviridae* (*n* = 829). The picornaviral reads could be further classified into five different genera of *Boosepivirus*, *Enterovirus*, *Kobuvirus*, *Bopivirus*, and *Erbovirus* (Fig. 1B). Sequences from potential eukaryotic viruses were analyzed further.

### Genome analysis of a caprine CRESS DNA virus

**Fig 2:**
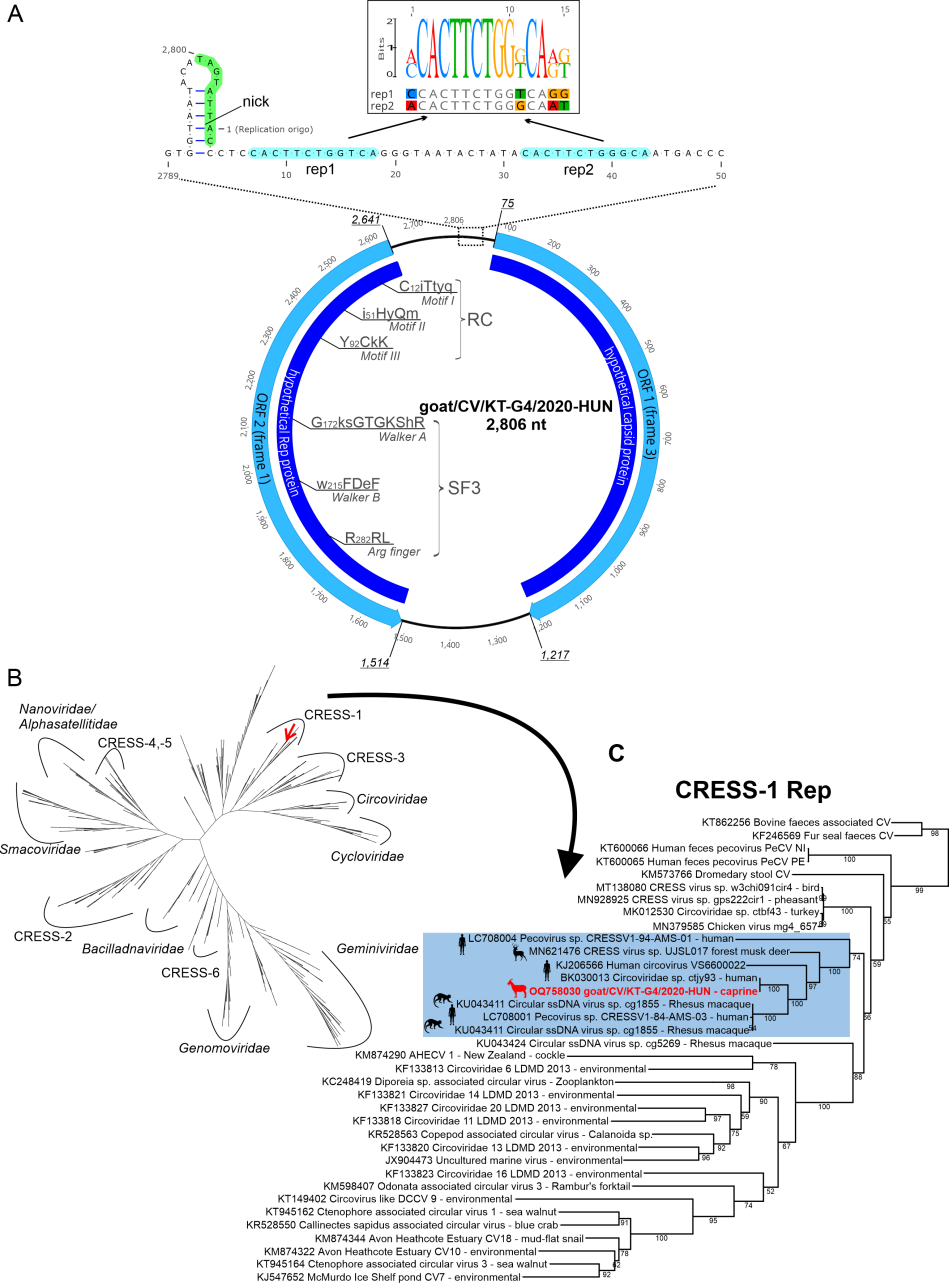
(**A**) A schematic genome map of CRESS DNA virus goat/CV/KT-G4/2020-HUN (OQ758030) including the features of predicted ORFs and encoded peptides as well as conserved amino acid motifs of replication protein (RC endonuclease and SF3 helicase domains). A genomic region which contains the presumed origin of viral replication was enlarged. The localizations of the highly conserved nonamer of the stem-loop (5′-TAGTATTAC-3′, with a green background including the site of Rep-mediated nick) and the adjacent short-sequence repeats (rep1, rep2, with blue background) were also marked. The alignment of the repeated sequences with a sequence logo was found above the structural model. Position 1 indicates the first residue of the genomic sequence of goat/CV/KT-G4/2020-HUN as uploaded to the GenBank database. (**B**) Maximum-likelihood phylogenetic tree generated from a protein alignment of the full-length Rep proteins of goat/CV/KT-G4/2020-HUN and *n* = 672 diverse CRESS DNA viruses from the study of Kazlauskas and co-workers (8). The alignment was supplemented with additional Rep sequences that showed the highest sequence similarities to the study strain from the results of BLASTp searches. The phylogenetic positions of the official (with *italics*) and proposed (CRESS-1–6) (8) CRESS DNA virus families are also indicated. The red arrow marked the localization of the study strain on the branch of CRESS-1 viruses. For a higher resolution version of the tree, see the supplementary figure (Fig. **S2**). (**C**) More detailed maximum-likelihood phylogenetic tree of the full-length Rep proteins of goat/CV/KT-G4/2020-HUN (in red) and related CRESS DNA viruses of the CRESS-1 lineage. The lineage of the study virus and closely related viruses from vertebrates with silhouettes of the hosts are marked with blue background. CV, circular virus. The maximum-likelihood trees were generated from the Clustal Omega alignments of Rep proteins using IQ-Tree web server, rtREV+G4+F model with 1,000 ultrafast bootstrap support.

The 2,806-nt-long complete genome of a circular CRESS DNA virus strain goat/CV/KT-G4/2020-HUN (OQ758030) was determined from mapped NGS reads using PCR and Sanger sequencing (Fig. S1; Fig. 2A). The acquired genome showed the highest sequence identity to a human *Circoviridae* sp. isolate ctjy93 (BK030013) from USA (Table 1). Two major ORFs (ORF1, 2) can be identified located in an antisense orientation (Fig. 2A). The ORF1 encodes a 380-aa-long protein which showed the highest sequence identity (97.03%) to the capsid protein of ctjy93. The second ORF encodes a 375-aa-long peptide which showed the highest sequence identities (99.32% and 98.8%) to Rep proteins of CRESS DNA viruses of ctjy93 and human circovirus VS6600032 (KJ206567), respectively. The conserved aa motifs of RC endonuclease (*Motifs I*, *II*, and *III*) and SF3 helicase domains (*Walker A/B*, *Arginine finger*) of Rep proteins are also recognizable (Fig. 2A). The *ori* region located between the initiation codons of both ORFs contains a presumed hairpin with a conserved nonamer (4, 32) followed by a short sequence repeat (Fig. 2A). The strain goat/CV/KT-G4/2020-HUN was clustered together with CRESS DNA viruses from humans and rhesus macaques and formed a distinct lineage in the currently unassigned “CRESS-1” virus group of circular ss DNA viruses in the Rep phylogenetic tree (Fig. 2B and C).

**TABLE 1:** Overview of the caprine viruses identified in this study and the most closely related strains together with taxonomical classifications and full-length nucleotide (nt) and amino acid (aa) identity percentages^*a*^

Identified viruses (accession no.)	Closest relatives (accession no.)	Classification of the closest relatives (*family/sub-family/genus/species*)	nt identity	aa identity
Goat/CV/KT-G4/2020- HUN (OQ758030)	Circoviridae sp. isolate ctjy93 (BK030013)	Unassigned CRESS DNA virus	99.32%	97.03% (Cap)/99.32% (Rep)
Goat tusavirus/KT-G5/2020/HUN (OL692339.2)	Goat tusavirus/KT-G5/2020/HUN (OL692339.1)	*Parvoviridae/Parvovirinae/Protoparvovirus/Tusavirus*	96%	100%
Goat/BooV/KT-FG3/2020- HUN (OQ758026)	Ovine PV. England/2004/E1028-04 (LR216006)	*Picornaviridae/Ensavirinae/Boosepivirus/Boosepivirus C*	73%	79%
Goat/EV/KT-FG3/2020- HUN (OQ758028)	Enterovirus sp. 344R-k141_987796 (MZ679311)	*Picornaviridae/Ensavirinae/Enterovirus/Enterovirus G*	78%	92%
Goat/KoV/KT-G4/2020- HUN (OQ758029)	Cattle/Kagoshima-2–24-KoV/2015/JPN (LC055960)	*Picornaviridae/Kodimesavirinae/Kobuvirus/Aichivirus D*	80%	85%
Goat/CapV/KT-G5/2020- HUN (OQ758027)	Bopivirus TCH6/USA/2013 (KM589358)	*Picornaviridae/Caphthovirinae/Bopivirus/Bopivirus A*	47%	39%
	Equine rhinitis B virus 1P1436/71 (X96871)	*Picornaviridae/Caphthovirinae/Erbovirus/Erbovirus A*	45%	35% (without the Leader)
Goat/MAstV/KT-G5/2020- HUN (OQ758025)	Caprine astrovirus G5.1 (MK404647)	*Astroviridae/ - /Mamastrovirus*	75%	90% (NSP1ab)/72% (Cap)

^*a*^ Cap, capsid; Rep, replication-associated protein; NSP1ab, non-structural protein 1ab; PV, picornavirus.

### Genome analysis of a caprine parvovirus

The majority (*n* = 6,602; 99.0%) of the identified *n* = 6,669 parvovirus-related reads could be mapped to the genome of *Protoparvovirus* sp. strain goat tusavirus/KT-G5/2020/HUN (OL692339.1) of genus *Protoparvovirus* subfamily *Parvovirinae* (Fig. S1) (11). The consensus sequence generated from the aligned reads is completely identical to the sequence of goat tusavirus/KT-G5/2020/HUN (OL692339.1) but 178 nt longer in which the region was verified by PCR and Sanger sequencing (data not shown).

### Overview of caprine picornaviruses

**Fig 3:**
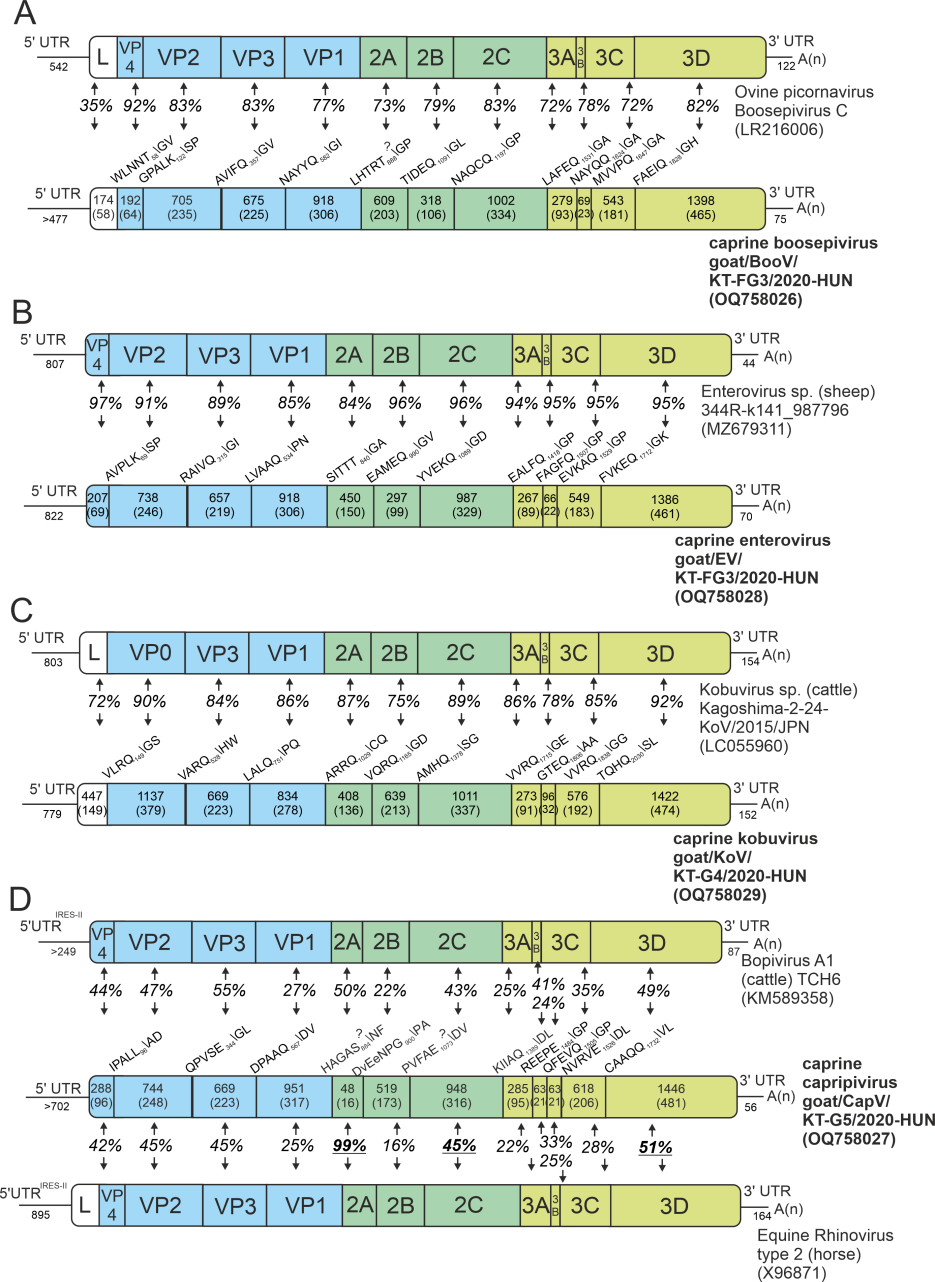
Schematic representations of genome layouts of identified caprine picornaviruses together with their closest relatives. Parts of the main P1, P2, and P3 genome regions are marked with light-blue, green, and yellow backgrounds, respectively. The positions and P5 – P2′ sequences of predicted proteolytic cleavage sites of the viral polyproteins of the study viruses are found above the borders of each genomic region. The pairwise amino acid sequence identity values (%) of different genomic regions of a study strain and its closest relative are found between the corresponding boxes. The nucleotide (upper numbers) and amino acid (lower numbers in brackets) lengths of different genomic regions are shown in each box. The pairwise identity values (%) between the corresponding genome parts of the study strain and Equine rhinovirus type 2 which are higher than the percentages between the corresponding genome parts of the study strain and Bopivirus A1 are underlined.

The complete genomes/CDS of a total of four different picornaviruses can be determined using the generated alignments of *n* = 829 picornaviral reads as templates for virus-specific primer designs and various RT-PCR, primer walking techniques, and Sanger sequencing (Fig. 1B; Fig. S1; Fig. 3).

### Genome analysis of caprine boosepivirus

**Fig 4:**
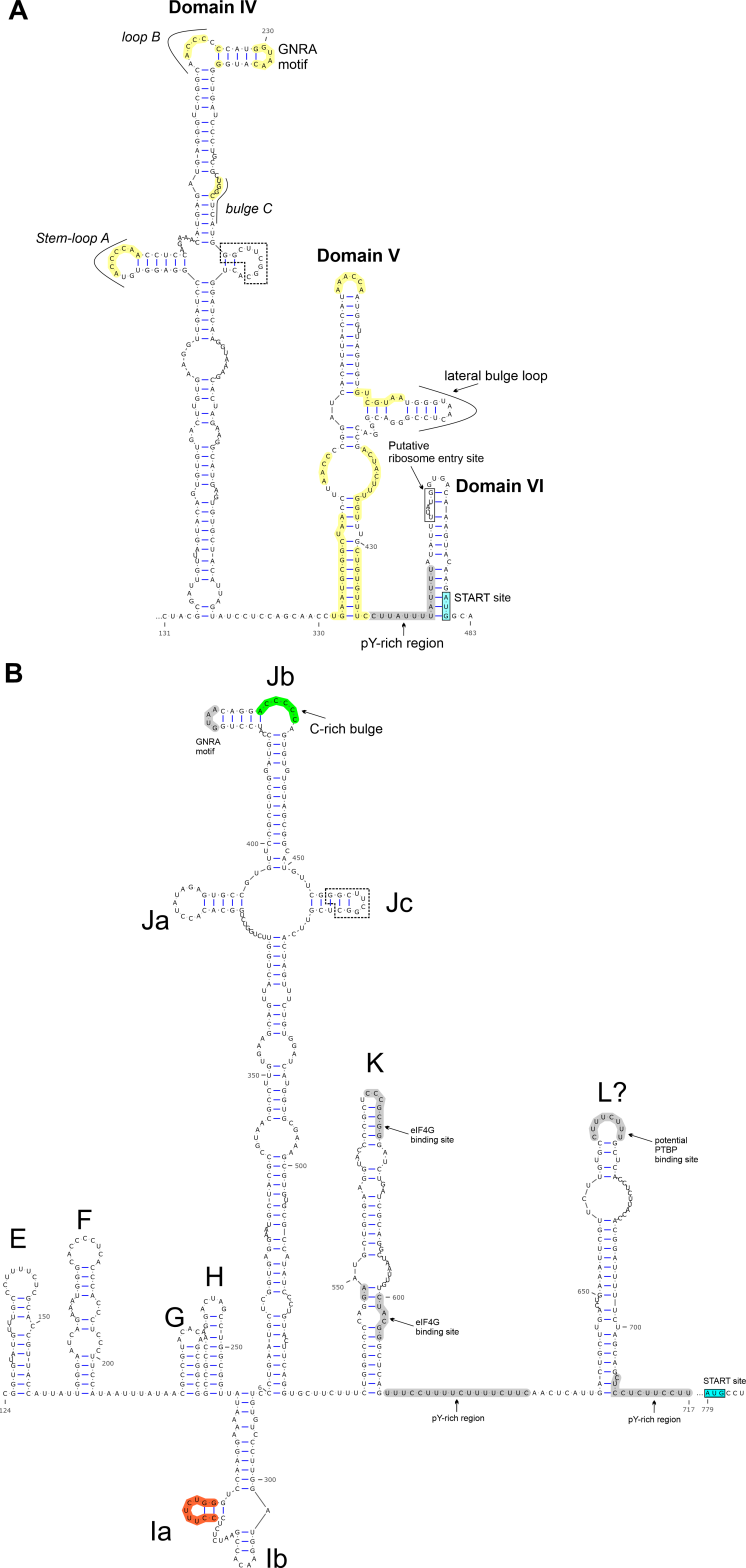
Predicted secondary RNA structures of presumed internal ribosomal entry sites (IRESes) of caprine boosepivirus strain goat/BooV/KT-FG3/2020-HUN (OQ758026) (**A**) and caprine kobuvirus strain goat/KoV/KT-G4/2020-HUN (OQ758029) (**B**). The main domains of IRESes (domains IV, V, and VI and domains E–L, respectively) were annotated similarly as previously proposed (panel A: 33; panel B: 42). The localizations of conserved motifs (stem-loops A, B, and bulge C, poly-pyrimidine-rich region/pY-rich regions, eIF4G, and pyrimidine tract binding protein/PTBP binding sites) of the main domains are also marked. Start codons are highlighted with a light-blue background. A highly conserved stem-loop region in the domain Jc of goat/KoV/KT-G4/2020-HUN (**B**) which could be also found in domain IV of type-I IRES of goat/BooV/KT-FG3/2020-HUN (**A**) is marked with dashed boxes. Nucleotide residues with a yellow background on panel A are identical between goat/BooV/KT-FG3/2020-HUN and the entero-/rhinovirus type-I IRESes (33, 34). Residues of a C-rich bulge which is uncommon in type-V IRESes but a characteristic motif of type-I IRESes (42, *loop B*) are marked with a green background on panel B. Residues of the Ia domain which are identical to the corresponding region of Aichivirus A1 (AB040749) are marked with an orange background.

The 7434-nt-long CDS of goat/BooV/KT-FG3/2020-HUN (OQ758026) showed the highest sequence identity to Boosepivirus C1 strain England/2004/E1028-04 from ovine (Table 1). The two viruses share the same genome layout with a homolog protease-type 2A (2A^Pro^ protease active site: G_1047_wCG, conserved aa-s are capitalized) and similar predicted cleavage sites (Fig. 3A). The 477-nt-long 5′UTR of goat/BooV/KT-FG3/2020-HUN is 62 nt shorter than the 5′UTR sequence of BooV-C1/England/2004/E1028-04 and shows 82% pairwise nt identity. Despite several attempts at different 5′RACE reactions (e.g., polyC, polyG, and polyA-tagging) the 5′ end of the study virus could not be extended further. The 5′UTR of goat/BooV/KT-FG3/2020/HUN was predicted to have a highly structured RNA conformation with characteristic main domains (domains IV, V, and VI) similar to those found in the Entero-/rhinoviral type-I IRESes (Fig. 4A). Besides the main structural resemblance, several conserved motifs of type-I IRESes like stem-loops A, B, and bulge C as well as the poly-pyrimidine-rich region and putative ribosome entry sites are also clearly recognizable (Fig. 4A).

The amino acid sequence identities between the corresponding genomic regions of goat/BooV/KT-FG3/2020-HUN and BooV-C1/England/2004/E1028-04 are varying between 35% (between leader peptides) and 92% (between VP4s) (Fig. 3A). The two strains show 21%, 19%, and 19% aa difference in the complete polyprotein, P1, and 2C+3CD proteins, respectively.

The 75-nt-long 3′UTR shows only 41% nt identity to the 122-nt-long corresponding genome part of BooV-C1/England/2004/E1028-04, and other sequences with higher identity have not been found by BLASTn searches. The goat/BooV/KT-FG3/2020-HUN is clustered together with the members of genus *Boosepivirus* in both 3D and P1 phylogenetic trees where the ovine picornaviruses of species *Boosepivirus C* are the closest relatives (Fig. 7 and 8A).

### Genome analysis of caprine enterovirus

The 7414-nt-long complete genome of goat/EV/KT-FG3/2020-HUN (OQ758028) showed the highest sequence identity to ovine Enterovirus G (EV-G) strain 344R-k141_987796 (MZ679311) (Table 1). The two viruses share the same genome layout with a similar Entero-/rhinoviral type-I IRES (data not shown), a homolog 2A^Pro^ (G_948_dCG), and similar predicted cleavage sites (Fig. 3B). The pairwise aa identities of the corresponding genomic regions of the two viruses are ranging between 84% (between the 2A peptides) and 97% (between VP4) while the immunodominant VP1 sequences show only 85% aa identity (Fig. 3B). Phylogenetically, the goat/EV/KT-FG3/2020-HUN is most closely related to ovine and caprine enteroviruses of species *Enterovirus G* but formed a separate lineage in both 3D^RdRp^ and VP1 phylogenetic trees (Fig. 7, Fig. 12A).

### Genome analysis of caprine kobuvirus

The 8446-nt-long complete genome and the 2504-aa-long viral polyprotein of caprine kobuvirus (CaKV) strain goat/KoV/KT-G4/2020-HUN (OQ758029) showed the highest sequence identity to bovine kobuvirus/Aichivirus D2 (AiV-D2) strain cattle/Kagoshima-2–24-KoV/2015/JPN (LC055960) of genus *Kobuvirus* species *Aichivirus D* (Table 1). The two viruses share the same genome layout with a homolog Hbox/NC-type 2A and similar predicted cleavage sites (Fig. 3C).

**Fig 5:**
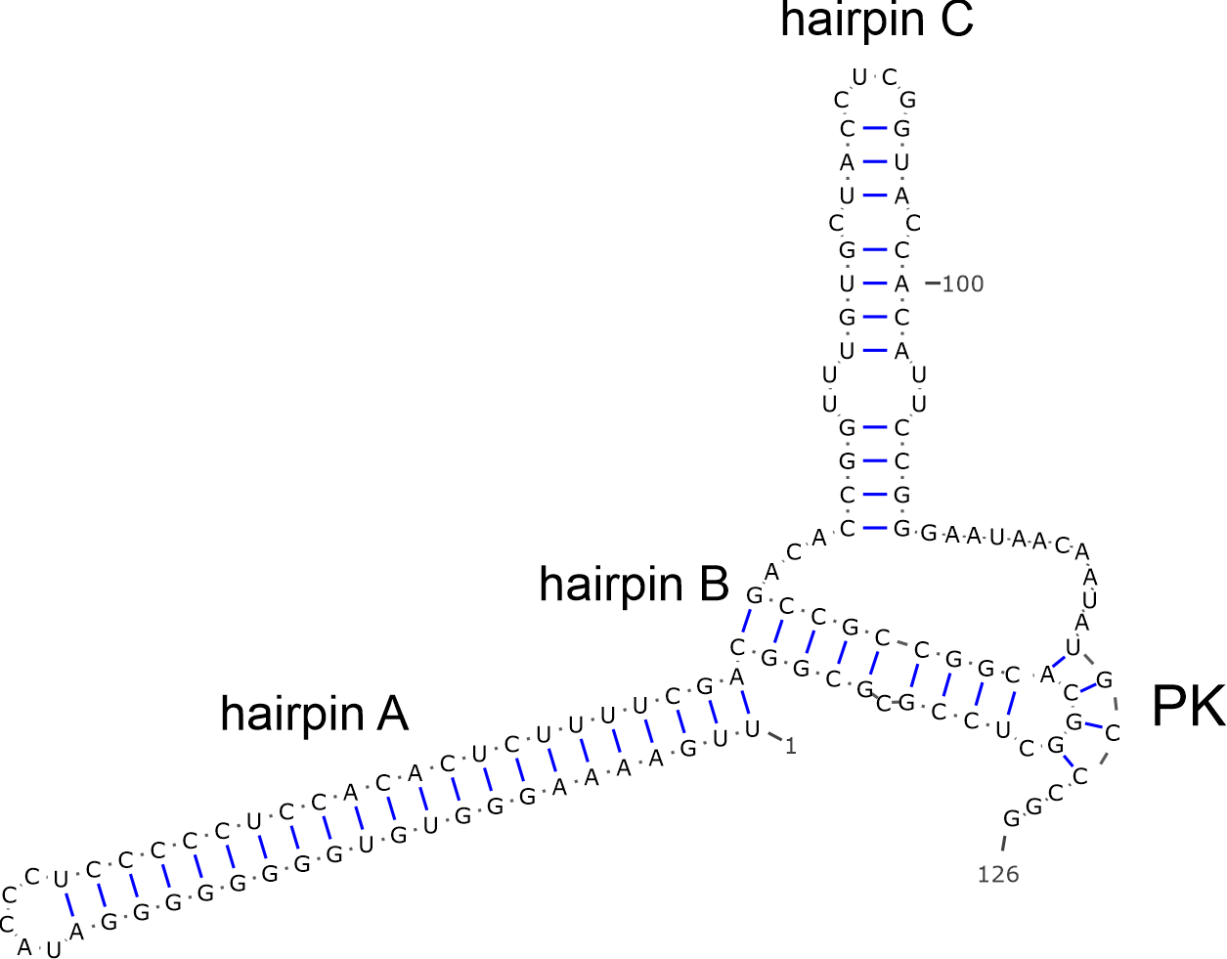
Predicted secondary RNA structure of 5′ terminal presumed origin of replication region (*ori*) of caprine kobuvirus strain goat/KoV/KT-G4/2020-HUN (OQ758029). The main domains of the *ori* were annotated similarly as previously proposed (35). PK, pseudoknot.

The 779-nt-long 5′UTR of goat/KoV/KT-G4/2020-HUN shows 80% nt identity to the corresponding genome part of cattle/Kagoshima-2–24-KoV/2015/JPN. The 5′UTR was predicted to have a 5′ terminal *ori* region with three adjacent hairpins and a pseudoknot (Fig. 5) followed by a highly structured region with multiple domains which resemble closely to the RNA architecture of Aichivirus/AV-like type-V IRESes (Fig. 4B). Besides structural similarities of the main domains (domains E–L), several conserved motifs of type-V IRESes like GNRA-tetraloop in the apex of domain J and eIF4G-binding motifs in domain K as well as poly-pyrimidine-rich regions are also clearly recognizable in the IRES of goat/KoV/KT-G4/2020-HUN (Fig. 4B). Interestingly, a unique C-rich bulge of domain Jb and a conserved hairpin of domain Jc which is identical to the stem-loop of domain IV of caprine boosepivirus goat/BooV/KT-FG3/2020-HUN was also identifiable (Fig. 4A and B). The aa sequence identities between the corresponding genomic regions of cattle/Kagoshima-2–24-KoV/2015/JPN and goat/KoV/KT-G4/2020-HUN are varying between 72% (between leader peptides) and 92% (between 3Ds) (Fig. 3C). The 152-nt-long 3′UTR of the study CaKV strain shows 80% nt identity to the 154-nt-long 3′UTR of cattle/Kagoshima-2–24-KoV/2015/JPN.

Phylogenetically, the goat/KoV/KT-G4/2020-HUN is most closely related to ovine and bovine kobuviruses of species *Aichivirus D* but formed a separate lineage in both 3D^RdRp^ and VP1 phylogenetic trees (Fig. 7 and 12B).

### Genome analysis of caprine erbo-/bopivirus-related picornavirus (capripivirus)

Sequence comparisons of the generated overlapping products from bopi- and erbovirus-read-based PCRs revealed complete nucleotide identities (Fig. S1) which suggests that these sequences originated from a single virus. Therefore, only a single, 7,400-nt-long complete coding sequence (the 5′ terminal end is probably missing) of goat/CapV/KT-G5/2020-HUN (OQ758027) could be determined from the mapped bopivirus-related and erbovirus-related reads (Fig. S1). The acquired CDS of the study virus which was provisionally designated as capripivirus [from the term caprine picornavirus (CapV)] showed the highest sequence identities to ERBV-1 strain P1436/71 (X96871) and BPV-A1 strain TCH6 (KM589358), as the closest relatives (Table 1). The genomic sequence of CapV was predicted to have a single ORF which starts with a translation-initiation site found in an optimal Kozak context (uCaAaGA_703_UGG, conserved nts are capitalized, and start codon is underlined). The ORF was flanked by a 5′ and a 3′UTR and a 3′ poly(A)-tail (Fig. 3D).

**Fig 6:**
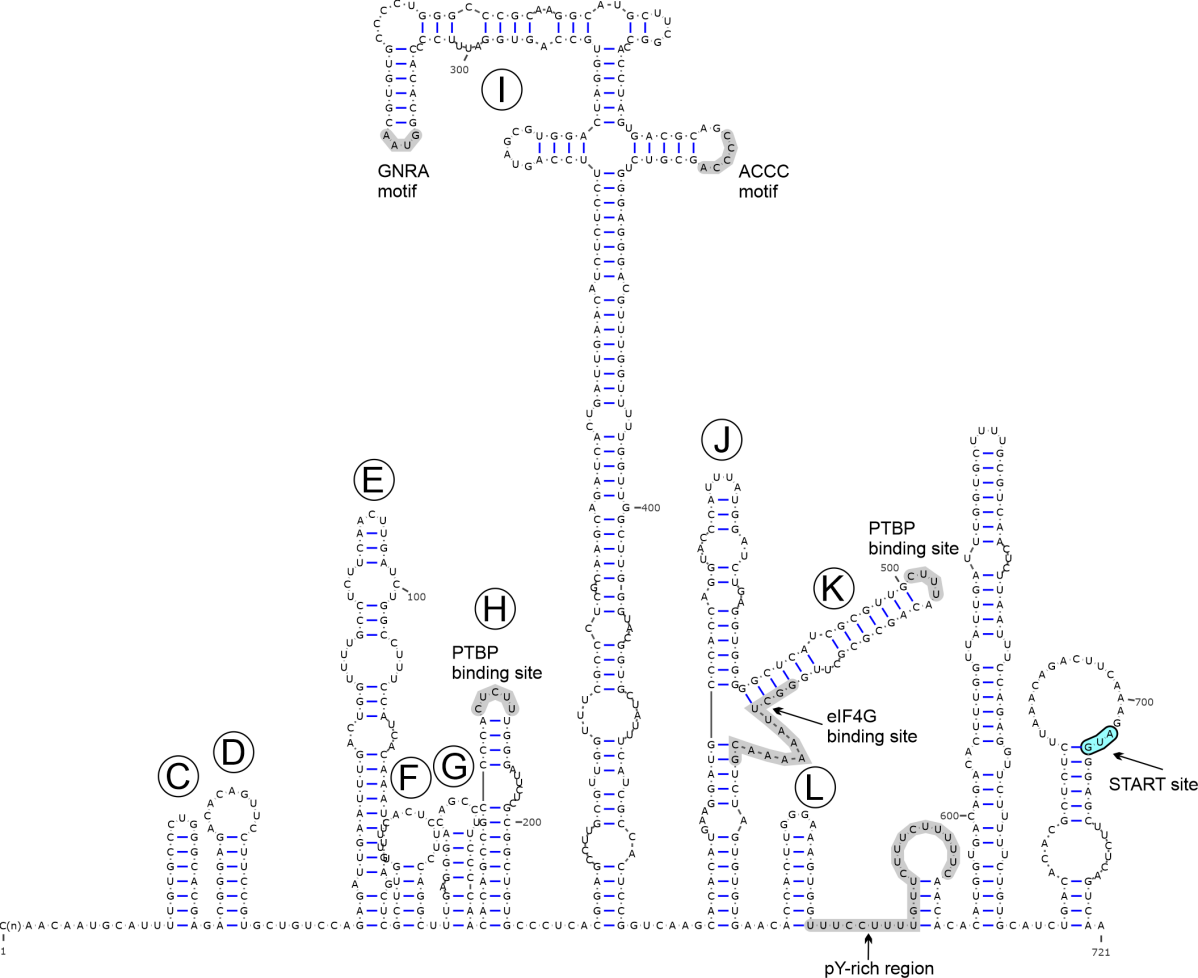
Predicted secondary RNA structure of the 5′UTR including the presumed internal ribosomal entry site (IRES) of caprine capripivirus strain goat/CapV/KT-G5/2020-HUN (OQ758027). The main domains were annotated from C to L similar to those previously proposed in the type-II IRES (37). The positions of conserved type-II IRES motifs are highlighted by a light-grey background. PTBP, pyrimidine tract binding protein; pY, poly-pyrimidine. The start codon was highlighted in light-blue.

The 702-nt-long 5′UTR of CapV is predicted to have a similar RNA architecture as found in type-II IRESes (Fig. 6) (33). Besides structural similarities with the main domains (domains C–L), several conserved motifs of type-II IRESes like GNRA and ACCC motifs in domain I and eIF4G-binding motif in domain K, potential pyrimidine tract binding protein (PTBP) binding sites in domains H and K, and poly-pyrimidine-rich regions (33, 36) are also clearly recognizable in the IRES (Fig. 6). Based on the absence of stem-loop structures corresponding to domains A and B of type-II IRES, the 5′ end of CapV is most likely incomplete.

The 2,213-aa-long viral polyprotein of CapV showed only 35% and 40% pairwise sequence identities to the polyproteins of ERBV-1/P1436/71 and BPV-A1/TCH6, respectively. Based on the predicted cleavage sites, the CapV have a similar Leaderless genome like bopiviruses but not like erboviruses (Fig. 3D). The 2A of CapV contains the conserved “Ribosome-skipping motif” of DxExNPGP (x means any aa) (38) (Fig. 3D). Interestingly, CapV contains two copies of a 21-aa-long 3B^VPg^ (3B1, 3B2; Fig. 3D) which shows 42% pairwise aa identity and completely identical N-terminal parts (G_1485/1506_PYSGF).

**Fig 7:**
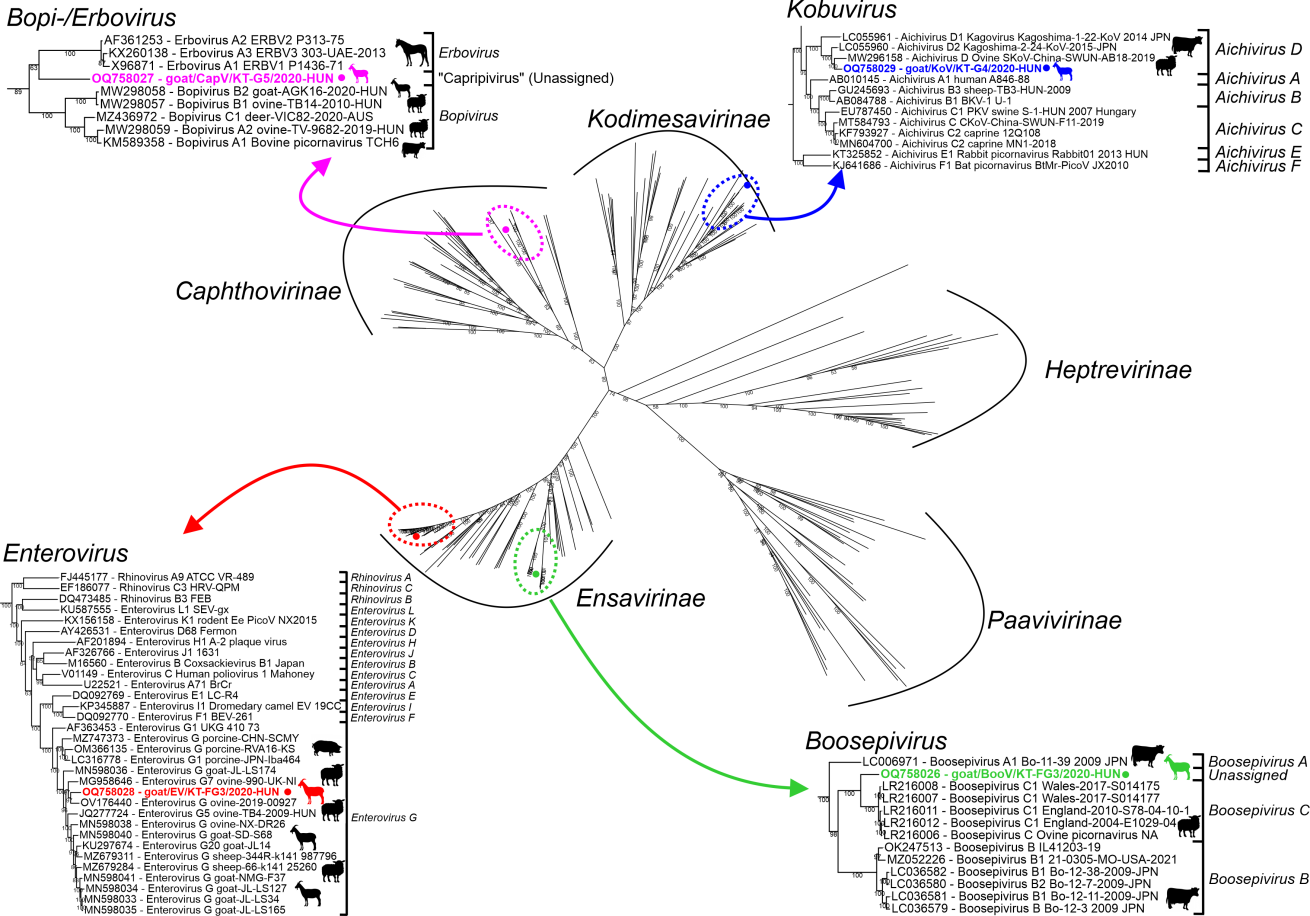
Phylogenetic analysis of the 3D nucleotide sequences of the study caprine picornaviruses (indicated with bold and distinct colors) and most similar sequences identified by BLAST searches together with other representative members of the *Picornaviridae* family. To help with orientation, the phylogenetic positions of the five subfamilies are marked with arches. The tree was generated from the codon-based nt alignment of *n* = 188 picornavirus sequences using IQtree with Ultrafast bootstrap (*n* = 1,000) and GTR+F+I+G4 model which was chosen automatically based on the BIC score. A higher resolution version of this tree with all labels is found in the supplementary figure (Fig. **S3**). The branches of genera *Enterovirus*, *Boosepivirus*, *Kobuvirus ,*and *Bopi-/Erbovirus* (labeled with colored dashed circles) which contain study strains and their closest relatives are enlarged. The hosts of the study strains and the closest relatives are marked with silhouettes.

**Fig 8:**
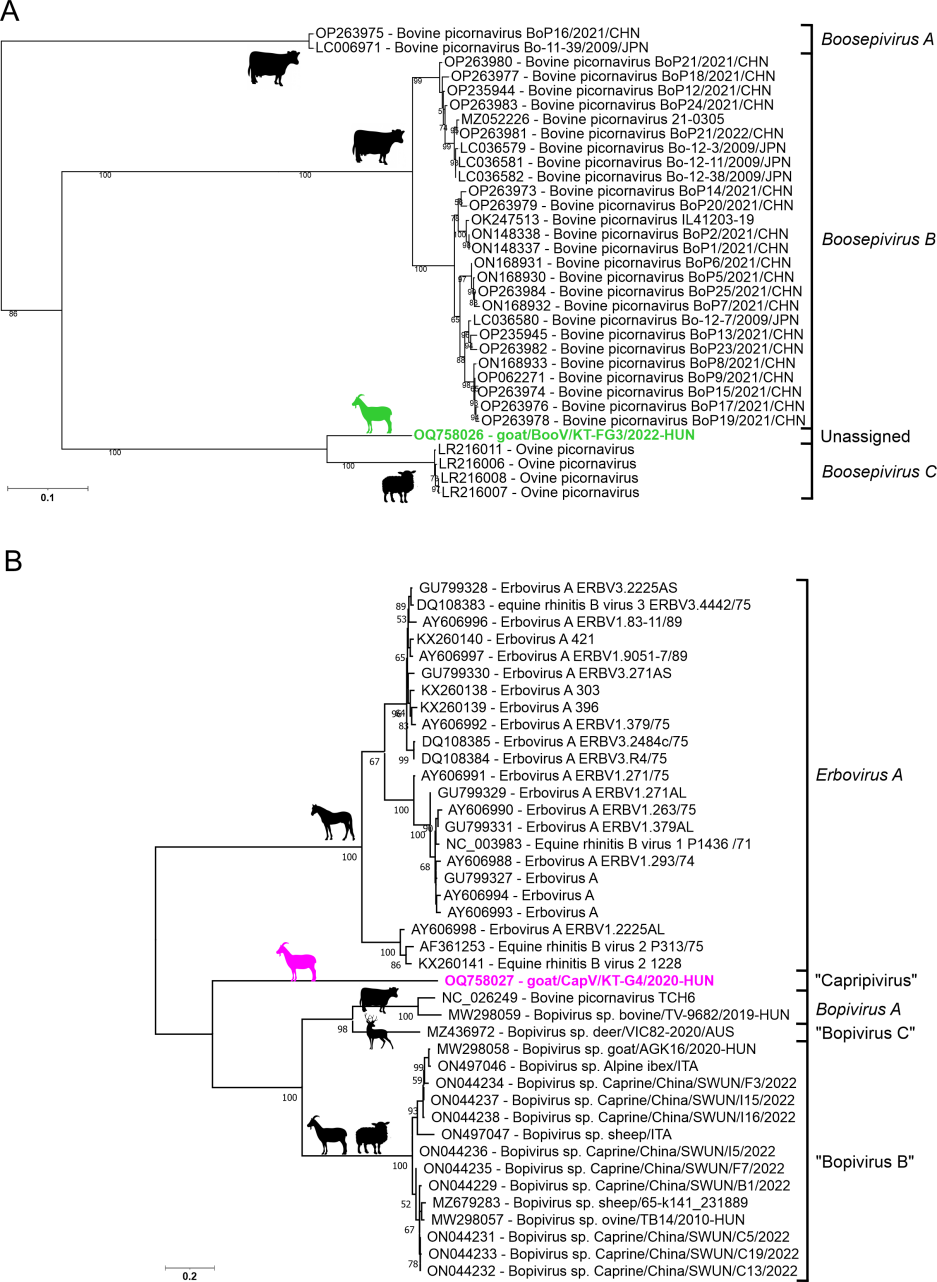
Phylogenetic analyses of P1 amino acid sequences of caprine boosepivirus goat/BooV/KT-FG3/2020-HUN (OQ758026, green) (**A**) and caprine capripivirus goat/CapV/KT-G5/2020-HUN (OQ758027, magenta) (**B**) together with their closest relatives. The Maximum likelihood trees were generated from complete P1 amino acid alignments using LG+F+G4 (**A**) and JTT matrix-based models (**B**) of MEGA ver 11 with 1000 bootstrap (BS) replicates. Only BS values higher than 50 were indicated. The positions of official (marked with italics) and proposed (marked with quotations) species of each genus are also indicated. The hosts of the strains are marked with silhouettes.

The pairwise comparisons of the corresponding genomic regions of CapV and either ERBV-1/P1436/71 or BPV-A1/TCH6 revealed that there are certain genomic regions like 2A, 2C, and 3D where CapV is more similar to ERBV-1/P1436/71 while in other regions, CapV is more resembling BPV-A1/TCH6 (Fig. 3D). The bopi- and erbovirus reads were mostly mapped to those genomic regions which are more similar to either BPV-A1/TCH6 or ERBV-1/P1436/71, respectively (Fig. S1). In the complete P1 region, the CapV shows 41% and 36% aa identities to BPV-A1/TCH6 and ERBV-1/P1436/71, respectively. The 56-nt-long 3′UTR of CapV is much shorter than the 87-nt- and 164-nt-long 3′UTRs of BPV-A1/TCH6 and ERBV-1/P1436/71 and shows only low (36% and 20%) sequence identities, respectively. The phylogenetic positions of goat/CapV/KT-G5/2020-HUN are also reflecting the results of sequence comparisons. On the 3D^RdRp^ phylogenetic tree, the CapV is clustered together with the erboviruses while in the P1 tree, it is more closely related to bopiviruses but formed separate lineages in both trees (Fig. 7 and 8B).

### Genome analysis of caprine astrovirus

**Fig 9:**
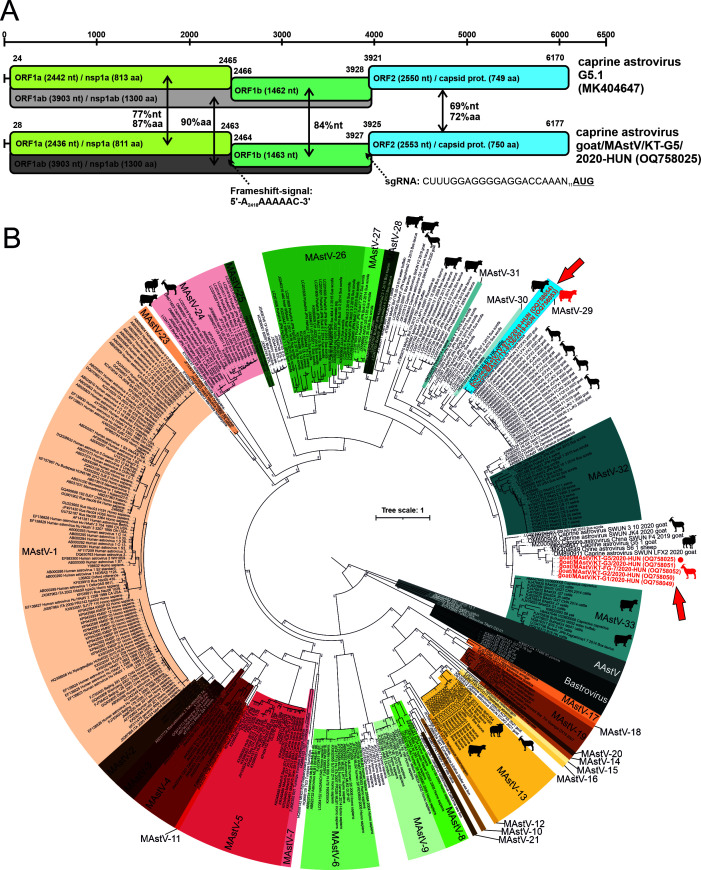
(**A**) Schematic genome maps with genomic features and identity values of the identified caprine astrovirus (CaAstV) strain goat/MAstV/KT-G5/2020-HUN together with its closest relative CaAstV strain G5.1. The ORF1a, ORF1b, and ORF2 genomic regions are marked with light-yellow, green, and blue backgrounds, respectively. nt, nucleotide; aa, amino acid; nsp1a/1ab, non-structural protein 1a/1ab. The positions and sequences of the conservative heptanucleotide frameshift signal near the ORF1a/1b junction region and the conservative subgenomic RNA (sgRNA) promoter/ORF2 initiation codon nt sequence were also shown in the figure. (**B**) Phylogenetic analysis of capsid amino acid sequences of AstVs identified in this study (written in red and marked with red arrows) together with *n* = 397 representative AstV and Bastrovirus sequences from (28, 28) and supplemented with the closest relatives of the study strains identified by BLAST search. The study CaAstV strain goat/MAstV/KT-G5/2020-HUN is also marked with a red circle. The maximum likelihood tree was generated with IQtree with JTT+I+G4+F model with Ultrafast bootstrap (*n* = 1,000). The caprine, ovine, and cattle hosts of the strains are marked with silhouettes.

The 6,250-nt-long complete genome of caprine astrovirus (CaAstV) strain goat/MAstV/KT-G5/2020-HUN (OQ758025) is predicted to have three ORFs (ORF1a, 1b, and ORF2), a conserved frameshift signal, and a subgenomic RNA (sgRNA) promoter similar as found in other MastVs (Fig. 9A). The ORF1a and 1b encode the non-structural proteins (NSP), the 811-aa-long NSP1a from ORF1a and the 1,300-aa-long NSP1ab from ORF1a and ORF1b (Fig. 9A). The ORF2 encodes the 750-aa-long viral capsid protein (Fig. 9A). The NSP1ab and the capsid proteins showed the highest sequence identities (p-dist 0.280) to caprine astrovirus G5.1 (MK404647) as the closest match (Fig. 9A; Table 1). Besides G5.1, the partial sequence (only capsid protein available) of caprine astrovirus strain SWUN/2-LFX2/2020 (OM890911) shows higher sequence identity (85%, p-dist: 0.150) to the capsid of the study CaAstV (Fig. 9A). Phylogenetic analysis of capsid proteins shows that goat/MAstV/KT-G5/2020-HUN is clustered together with multiple other CaAstVs including G5.1 and SWUN/2-LFX2/2020 and a single ovine AstV, and together, they formed a distinct lineage (Fig. 9B).

### Epidemiological investigations of caprine RNA viruses

**Fig 10:**
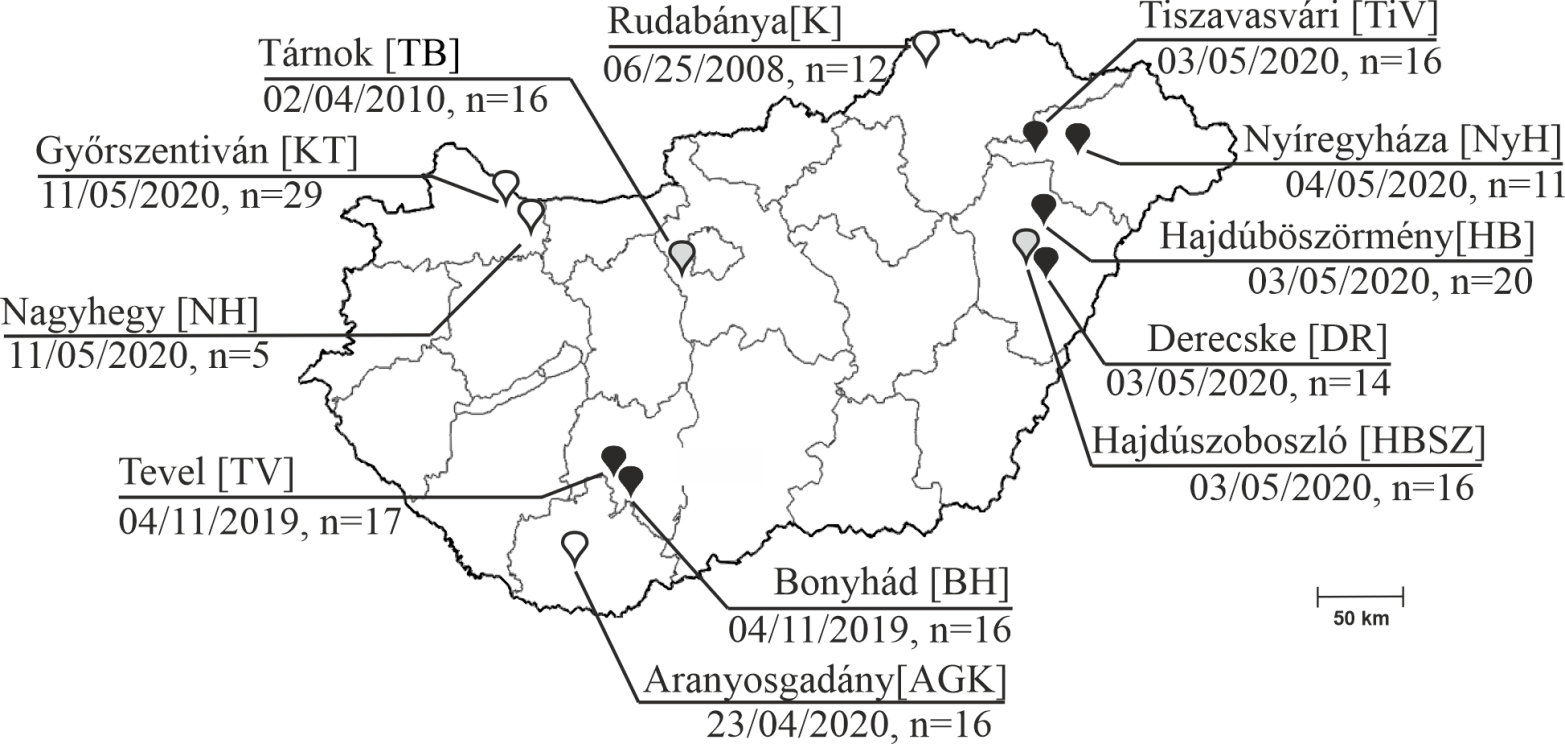
Localizations of the investigated farms [farm IDs] in Hungary together with collection dates and numbers of fecal samples (∑=188) which were collected from caprine (empty markers), ovine (gray markers), and cattle (black markers). The map was created using Corell Draw software.

For epidemiological investigations of the identified RNA virus groups, multiple “in-house” developed singleplex SYBRg-based qPCR assays (Boosepivirus C/BooV-C, Enterovirus G/EV-G, Aichivirus D/AiV-D, “capripivirus”/CapV, and goat/MAstV/KT-G5/2020-HUN-related MAstVs) were applied (Table S1 and S2; Fig. S4). The assays were used for screening additional enteric samples originating from *n* = 62 caprine, *n* = 32 ovine, and *n* = 94 cattle from different farms in Hungary (Fig. 10). A total of 132/188 (70.2%) samples were positive for at least one of the qPCR assays. The overall and age group-specific prevalence data can be found in Tables 2 and 3 while the co-infection rates of the investigated RNA viruses were summarized in Table 4. MAstVs were the most common viruses found in co-infections together with either AiV-D in caprine (10/62, 16.1%) and cattle (23/94, 24.5%) or EV-G viruses in ovine (14/32, 43.8%), respectively (Table S3). Quadruple and quintuple infections were found only among the youngest (group I) less than 2-month-old animals (Table 4; Table S3).

**TABLE 2:** SYBRg-qPCR-based prevalence data of Boosepivirus C (BooV-C), Enterovirus G (EV-G), Aichivirus D (AiV-D), Capripivirus (CapV), and goat/MAstV/KT-G5/2020-HUN-related astroviruses (MAstV) in enteric samples of different hosts which belong to three age groups (I, II, and III) from various Hungarian farms (farm identifications are in square brackets)^*a*^

Host species	Farm location [Farm ID]	Management type	No. of positive fecal samples/total by age groups*															
			<2 months					(II) 2–12 months					(III) >12 months					
			BooV-C	EV-G	AiV-D	CapV	MAstV	BooV-C	EV-G	AiV-D	CapV	MAstV	BooV-C		EV-G	AiV-D	CapV	MAstV
**Caprine**	Aranyosgadány [AGK]	Extensive	–	–	–	–	–	0/8	1/8	0/8	1/8	0/8	0/8		1/8	1/8	0/8	0/8
	Győrszentiván [KT]	Intensive	5/9	9/9	4/9	4/9	8/9	3/10	0/10	5/10	8/10	8/10	2/10		4/10	5/10	1/10	4/10
	Nagyhegy [NH]	Extensive	–	–	–	–	–	–	–	–	–	–	0/5		2/5	0/5	0/5	0/5
	Rudabánya [K]	Extensive	–	–	–	–	–	0/12	3/12	0/12	0/12	0/12	–		–	–	–	–
**Ʃ=**			**5/9** (**55.5%**)	**9/9** (**100%**)	**4/9** (**44.4%**)	**4/9** (**44.4%**)	**8/9** (**88.9%**)	**3/30** (**10.0%**)	**4/30** (**13.3%**)	**5/30** (**16.7%**)	**9/30** (**30.0%**)	**8/30** (**26.7%**)	**2/23** (**8.7%**)		**7/23** (**30.4%**)	**6/23** (**26.1%**)	**1/23** (**4.4%**)	**4/23** (**17.4%**)
**Ovine**	Hajdúszoboszló [HBSz]	Extensive	0/8	7/8	5/8	1/8	4/8	0/5	5/5	2/5	4/5	4/5	0/3		2/3	2/3	0/3	1/3
	Tárnok [TB]	Extensive	0/16	11/16	9/16	1/16	8/16	–	–	–	–	–	–		–	–	–	–
**Ʃ=**			**0/24** (**0.0%**)	**18/24** (**75.0%**)	**14/24** (**75.0%**)	**2/24** (**8.3%**)	**12/24** (**50.0%**)	**0/5** (**0.0%**)	**5/5** (**100%**)	**2/5** (**40.0%**)	**4/5** (**80.0%**)	**4/5** (**80.0%**)	**0/3** (**0.0%**)		**2/3** (**66.7%**)	**2/3** (**66.7%**)	**0/3** (**0.0%**)	**1/3** (**33.3%**)
**Cattle**	Hajdúböszörmény [HB]	Intensive	0/18	0/18	3/18	0/18	9/18	–	–	–	–	–	0/2		1/2	1/2	0/2	2/2
	Nyíregyháza [NyH]	Intensive	0/5	0/5	1/5	0/5	4/5	0/4	0/4	4/4	0/4	2/4	0/2		0/2	2/2	0/2	1/2
	Derecske [DR]	Extensive	0/2	0/2	2/2	0/2	2/2	0/1	0/1	1/1	0/1	0/1	0/11		0/11	7/11	0/11	5/11
	Tiszavasvári [TiV]	Intensive	0/8	0/8	1/8	0/8	3/8	0/7	0/7	6/7	0/7	6/7	0/1		0/1	0/1	0/1	0/1
	Bonyhád [BH]	Intensive	0/16	0/16	3/16	0/16	10/16	–	–	–	–	–	–		–	–	–	–
	Tevel [TV]	Intensive	0/17	0/17	3/17	0/17	13/17	–	–	–	–	–	–		–	–	–	–
**Ʃ=**			**0/66** (**0.0%**)	**0/66** (**0.0%**)	**13/66** (**19.7%**)	**0/66** (**0.0%**)	**41/66** (**62.1%**)	**0/12** (**0.0%**)	**0/12** (**0.0%**)	**11/12** (**91.7%**)	**0/12** (**0.0%**)	**8/12** (**66.7%**)	**0/16** (**0.0%**)		**0/16** (**0.0%**)	**10/16** (**62.5%**)	**0/16** (**0.0%**)	**8/16** (**50.0%**)

^*a*^ mo, month old. Management type—Extensive: animals of different ages were released freely during the daytime and were housed together indoors at night. Intensive: animals were kept in closed indoor stockyards where the goats were held together while the cattle were kept in separate pens; animals of similar age groups were held together. The total number of positives with percentages was written in bold. –, no sample available.

**TABLE 3:** Overall prevalence of Boosepivirus C (BooV-C), Enterovirus G (EV-G), Aichivirus D (AiV-D), Capripivirus (CapV), and goat/MAstV/KT-G5/2020-HUN-related astroviruses (MAstV) in enteric samples of different hosts based on the results of SYBRg-qPCR-based screening assays^*a*^

Host species	BooV-C	EV-G	AiV-D	CapV	MAstV
**Caprine**	10/62 (16.1%)	**20/62** (**32.3%**)	15/62 (24.2%)	14/62 (22.6%)	**20/62** (**32.3%**)
**Ovine**	0/32 (0.0%)	**25/32** (**78.5%**)	18/32 (56.3%)	6/32 (18.8%)	17/32 (53.1%)
**Cattle**	0/94 (0.0%)	0/94 (0.0%)	34/94 (36.2%)	0/94 (0.0%)	**57/94** (**60.6%**)

^*a*^ The highest total numbers of positives with percentages are written in bold.

**TABLE 4:** Summary of co-infection rates (with percentages) of the four RNA viruses (Boosepivirus C, Enterovirus G, Aichivirus D, Capripivirus, and goat/MAstV/KT-G5/2020-HUN-related astroviruses) in enteric samples of different hosts based on the results of SYBRg-qPCR-based screening assays^*a*^

Host species	Age group	Dual infections	Triple infections	Quadruple infections	Quintuple infections	Total co-infections
**Caprine**	I	3/9	2/9	**2/9**	**2/9**	9/9
		(33.3%)	(22.2%)	(**22.2%**)	(**22.2%**)	(100%)
	II	3/30	6/30	0/30	0/30	9/30
		(10.0%)	(20.0%)	(0.0%)	(0.0%)	(30.0%)
	III	4/23	2/23	0/23	0/23	6/23
		(17.4%)	(8.7%)	(0.0%)	(0.0%)	(26.1%)
	Σ	10/62	10/62	2/62	2/62	24/62
		(16.1%)	(16.1%)	(3.2%)	(3.2%)	(38.7%)
**Ovine**	I	9/24	6/24	**1/24**	0/24	16/24
		(37.5%)	(25.0%)	(**4.2%**)	(0.0%)	(66.7%)
	II	0/5	5/5	0/5	0/5	5/5
		(0.0%)	(100%)	(0.0%)	(0.0%)	(100%)
	III	1/3	1/3	0/3	0/3	2/3
		(33.3%)	(33.3%)	(0.0%)	(0.0%)	(66.7%)
	Σ	10/32	12/32	1/32	0/32	23/32
		(31.3%)	(37.5%)	(3.1%)	(0.0%)	(71.9%)
**Cattle**	I	11/66	0/66	0/66	0/66	11/66
		(16.7%)	(0.0%)	(0.0%)	(0.0%)	(16.7%)
	II	8/12	0/12	0/12	0/12	8/12
		(66.7%)	(0.0%)	(0.0%)	(0.0%)	(66.7%)
	III	4/16	0/16	0/16	0/16	4/16
		(25.0%)	(0.0%)	(0.0%)	(0.0%)	(25.0%)
	Σ	23/94	0/94	0/94	0/94	23/94
		(24.5%)	(0.0%)	(0.0%)	(0.0%)	(24.5%)

^*a*^ Quadruple and quintuple infections are written in bold. Details about age groups are found in the Materials and Methods section and in Table 2.

A total of 54 selected samples with the lowest Ct (at least two samples/farm/host) were sequenced and compared with each other and the sequences of the GenBank using BLASTn (Table S4). The acquired partial RdRp sequences show ≥84/≥98% (CapV, *n* = 4 sequences), ≥99/100% (BooV-C, *n* = 2), ≥82/≥93% (EV-G, *n* = 11), ≥81/≥88% (AiV-D, *n* = 19), and ≥73/≥81% (MAstV, *n* = 18) nt/aa% pairwise sequence identities compared with each other. All the determined sequences from the qPCR assays showed the highest sequence identities in BLASTn searches to viruses belonging to those virus groups for which the corresponding assay was developed (Table S4).

**Fig 11:**
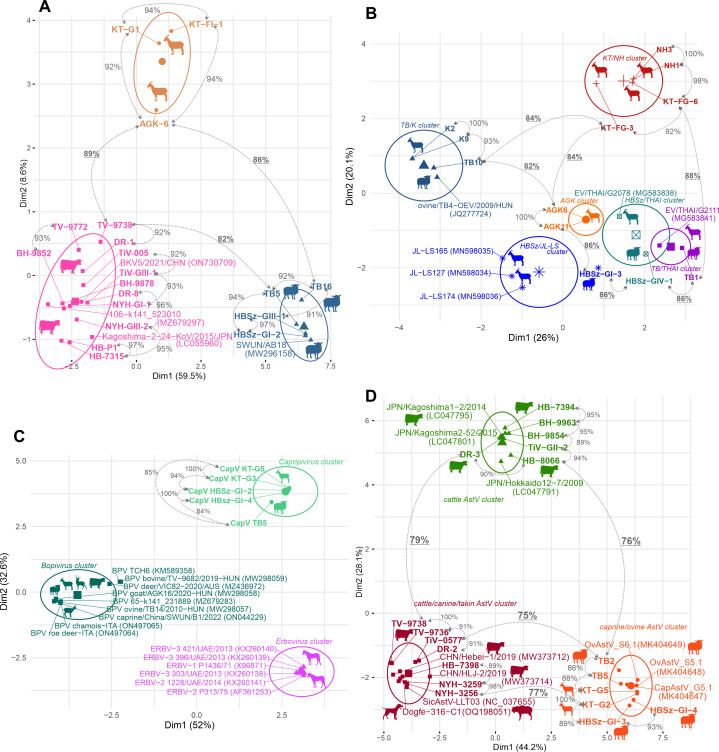
K-means cluster analyses of the nucleotide alignments of the determined partial RNA-dependent RNA polymerase (RdRp) sequences from the qPCR assays (only sequence IDs are presented in bold) together with selected closely related sequences identified by BLASTn searches (Aichivirus D: panel A, Enterovirus G: panel B, Capripivirus: panel C and Mamastrovirus: panel D). Details of the sequence IDs (including lengths, strain names, origins, and accession numbers) are found in Table S4. Sequences from the same cluster are marked with the same color. The hosts of the sequences are illustrated with silhouettes. Pairwise sequence identity values (%) are marked between sequences connected by dotted double arrows. Inter-cluster identity values (selected pairwise identities between sequences from different clusters) are underlined.

K-means cluster analyses were conducted on the nt alignments of the determined RdRp sequences of AiV-D, EV-G, CapV, and MAstV together with selected most similar viruses identified by BLASTn searches (Fig. 11; Table S4). The AiV-D sequences from the same host were grouped together and separated into three clusters (Fig. 11A). Sequences from the same cluster/host show 91%–97% pairwise nt identity and sequences between clusters/from different hosts show ≤89% nt identity. (Fig. 11A). In case of EV-G, a total of six different clusters could be distinguishable (Fig. 11B). There are clusters where only sequences from caprine are present (AGK cluster or KT/NH cluster), but multiple (*n* = 4) mixed caprine/ovine clusters are also identifiable with high intra-cluster sequence identities (Fig. 11B). There are ovine farms (Farm TB and HBSz) where multiple EV-G strains (TB1 and TB10, HBSz-GIV-1, and HBSz-GI-3) of different clusters were simultaneously present (Fig. 11B). The CapV RdRp sequences from caprine and ovine hosts are clustered together and separated from the erbo- and bopivirus clusters (Fig. 11C). Capripivirus sequences from the same farms (e.g., caprine KT and ovine HBSz farms) are identical to each other. Capripivirus RdRp-s from ovine farm HBSz show 94% nt/98% aa identities (only 1/82 aa difference) to CapV sequences from caprine which originated from a farm (Farm KT) which is c.a. 340 km away (Fig. 10). The CapV strain from Farm TB shows only 84% and 85% nt identities to the other CapV sequences (Fig. 11C). The MAstV RdRp sequences are separated into three distinct clusters, one of them contains only cattle-origin AstVs (cattle AstV cluster) while the two are mixed clusters which contain AstV sequences from different hosts (cattle/canine/takin and caprine/ovine AstV) (Fig. 11D). There are sequences from cattle farms DR, TiV, or HB where diverse AstV strains belonging to different clusters are present (Fig. 11D).

**Fig 12:**
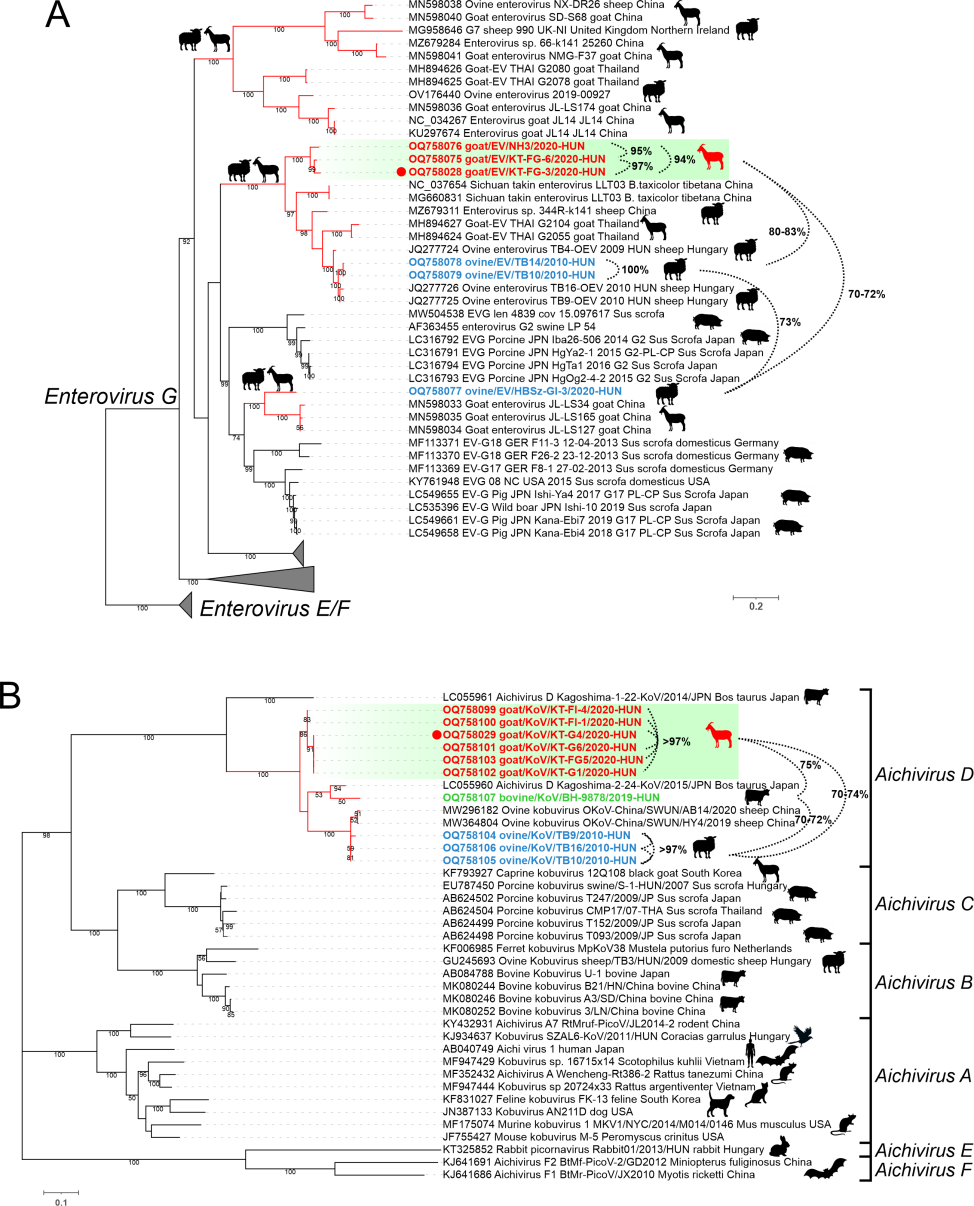
Maximum likelihood phylogenetic trees of VP1 amino acid (aa) sequences of study enteroviruses (**A**) and kobuviruses (**B**) and its closest relatives identified by BLASTp searches. The trees were generated from the separate alignments of 306-aa-long complete (**A**) and 95/108-aa-long partial (**B**) VP1 sequences using LG+F+G4 (**A**) and JTT matrix-based models (**B**) using IQtree with Ultrafast bootstrap (*n* = 1,000). Only BS values higher than 50 were indicated. Enterovirus G sequences unrelated to the study strains were collapsed. Study sequences identified from ovine, caprine, and cattle were marked with blue, red, and green, respectively. Study strains with determined complete genomes are marked with red dots. Percentages are pairwise aa identity values between sequences connected by dotted lines. Branches of viruses from various ruminant species including the study strains are drawn with red lines. The lineages of caprine entero- and kobuviruses are highlighted with a green background. The hosts of the various strains are marked with silhouettes.

Partial capsid sequences could be determined only in 8/94, 10/67, 5/46, 2/20, and 1/10 of MAstV, AiV-D, EV-G, CapV, and BooV-C qPCR-positive samples, respectively. The 795-nt-long CapV (OQ758062, OQ758063) and 816-nt-long BooV-C (OQ758057) capsid sequences show 99% nt and 100% aa identities with the corresponding genome parts of the index CapV or BooV-C strains; therefore, no further phylogenetic analyses were conducted on those sequences. Enteroviral VP1 capsid sequences could be determined only from two caprine (Farm KT and NH) and ovine farms (Farm TB and HBSz). The determined *n* = 5 capsid sequences of EV-G strains from the same farm show high pairwise identities (>97%), and they are clustered together and formed distinct lineages in the VP1 amino acid phylogenetic tree (Fig. 12A). Kobuviral VP1 sequences could be determined only from a single caprine (Farm KT), ovine (Farm TB), and cattle (Farm BH) farm. The phylogenetic and BLAST-based sequence analyses of capsid sequences indicate that all the identified KV strains belong to the species *Aichivirus D* and formed three host-specific lineages (Fig. 12B). Astrovirus capsid sequences could be determined only from a single caprine farm (Farm KT, *n* = 4 sequences) and cattle farm (Farm TV, *n* = 2 sequences). All sequences from caprine farm KT are identical to each other and clustered together with the index CaAstV strain goat/MastV/KT-G5/2020-HUN while the sequences from cattle are distant from the CaAstVs of this study and grouped with a cattle AstV strain B76-HK (HQ916316) (Fig. 9B).

## DISCUSSION

Based on the results of sequence and phylogenetic analyses, the identified CRESS DNA virus strain goat/CV/KT-G4/2020-HUN (OQ758030) most likely belongs to a currently unassigned viral family (“CRESS-1”) of order *Cirlivirales* and clustered together with certain human and macaque viruses. The closest relatives of goat/CV/KT-G4/2020-HUN are the strain ctjy93 (BK030013) which was originally identified from a fecal sample of a 23-year-old female and the strain VS6600032 (KJ206567) which was detected from an enteric sample from a 90-year-old patient (39, 40). The high sequence identities between these human and goat CRESS DNA viruses suggest the potential zoonotic (or reverse-zoonotic) transmission capabilities of these viruses with currently unknown pathogenic potential.

Interestingly, the genome of caprine tusavirus/KT-G5/2020/HUN (OL692339.2) of family *Parvoviridae* determined in this study was 178 nt longer than the previous version of this sequence (OL692339.1), 37 nt longer than its closest relative of human tusavirus 1 strain Tu491 (KJ495710), and it is so far the longest/most complete known tusavirus sequence. For detailed genomic, epidemiological, and phylogenetic analyses of caprine and closely related ovine tusaviruses, see our previous report (11).

Based on the results of genomic and phylogenetic analyses, the strain goat/BooV/KT-FG3/2020-HUN most likely belongs to the genus *Boosepivirus*. Besides cattle and ovine hosts, the goat/BooV/KT-FG3/2020-HUN is the first member of the genus *Boosepivirus* identified from caprine which has similar type-I IRES as the cattle BooV-A & B strains (24) but with the first complete structural model of the full-length IRES. The goat/BooV/KT-FG3/2020-HUN shows the closest relationship to the recently identified ovine BooVs of species *Boosepivirus C* (25). According to the species demarcation criteria of BooVs (30%, 40%, and 35% aa difference in the complete polyprotein, P1, 2C+3 CD, respectively, https://ictv.global/report/chapter/picornaviridae/picornaviridae/boosepivirus), the study caprine BooV most likely belongs to the species *Boosepivirus C* as a novel genotype (“boosepivirus C2”). The neurotropic potential (or inter-species transmission capability) of the identified caprine BooV is currently unknown but should be considered because of the close relationship to neurotrophic BooVs which were detected in brain and spinal cord samples of lambs with non-suppurative encephalomyelitis (25).

The identified goat/EV/KT-FG3/2020-HUN (OQ758028) strain most likely belongs to the species *Enterovirus G* together with the majority of known caprine and ovine EVs (21, www.picornaviridae.com). Based on the sequence-based genotype determination criteria (VP1 aa sequence divergence between different EV types should be >12%) (41), the caprine EV-G strain goat/EV/KT-FG3/2020-HUN could be a novel genotype in species *Enterovirus G* tentatively designated as “EV-G24”. Enterovirus G viruses were detectable by qPCR in multiple caprine and ovine samples of all ovine and caprine farms and found frequently in co-infections, but undetectable in cattle samples. VP1 capsid sequences could only be determined from c.a. 10% of the EV-G qPCR-positive samples from two caprine (Farm KT, NH) and two ovine farms (Farm TB and HBSz). Based on the high sequence identities (>94% aa), the identified EV-G strains from the farm KT and farm NH belong to the same type of “EV-G24” as the index caprine EV-G strain (goat/gEV/KT-FG3/2020-HUN). The ovine EV-G strains from Farm TB most likely belong to the EV-G5 type as the other previously described ovine EV-G strains of the same farm (42). Based on considerable sequence divergence (15% aa) in the VP1, the ovine/EV/HBSz-GI-3/2020-HUN is most likely a founding member of a novel genotype, tentatively designated as “EV-G25.” Based on the results of 3D^RdRp^ cluster- and VP1 capsid-based phylogenetic analyses, the identified EV-G viruses circulating in caprine and ovine farms in Hungary are remarkably diverse; multiple types of EV-G could be present simultaneously in the populations with possible interspecies transmissions of these viruses between ovine and caprine similar as suggested before (21).

The strain goat/KoV/KT-G4/2020-HUN most likely belongs to species *Aichivirus D* of the genus *Kobuvirus* with the highest sequence identity to a cattle kobuvirus from Japan (43). Until now, members of *Aichivirus D* with complete genomes were described only from cattle and ovine hosts (43, 44). Based on the considerable sequence difference and distinct phylogenetic position, the goat/KoV/KT-G4/2020-HUN could belong to a novel genotype in species *Aichivirus D*. The 5′ terminal region of the genome of goat/KoV/KT-G4/2020-HUN predicted to has an origin of replication (*ori*) region like other kobuviruses (35, 44). According to our current knowledge, the secondary RNA structure of an AiV-D IRES was not analyzed before. The structural analyses of 5′UTR indicated that goat/KoV/KT-G4/2020-HUN has a type-V-like IRES similar to the members of *Aichivirus A* and *B* (35, 45), but with some characteristic differences. The apical part of domain J (Jb) contains a C-rich bulge which was not identifiable in other J domains of type-V IRESes; moreover, the absence of this C-rich bulge was previously known to be a characteristic feature of all type-V IRESes (45). The apical C-rich bulge is a characteristic feature of domain IV of type-I IRESes (which domain has strong structural similarity to domain J of type-V IRESes) (45) where it was found to have a strong effect on the efficiency of *in vitro* RNA translation of type-I IRESes by serving as a poly(rC) binding protein 2 (PCBP2)—a cellular IRES trans-acting factor (ITAF)—binding site (34). The presence of this presumed PCBP2 binding site in the IRES of goat/KoV/KT-G4/2020-HUN could suggest the use of an alternative type-I IRES-like translation regulation mechanism of certain versions of type-V-like IRESes (34, 46). Furthermore, a short stem-loop of domain Jc of goat/KoV/KT-G4/2020-HUN was completely identical to a stem-loop of domain IV of caprine boosepivirus strain goat/BooV/KT-FG3/2020-HUN (Fig. 4A and B) which could be an example of the modular exchange of certain IRES elements between different co-infecting viruses similar as suggested before (13, 14).

From the investigated picornaviruses, AiV-D was overall the most prevalent; it was detectable in multiple samples of caprine, ovine, and cattle hosts and frequently found in co-infections in nearly all farms. VP1 sequences could be determined only from a minority of AiV-D-positive samples (*n* = 9/67) which originated from a single caprine (Farm KT), ovine (Farm TB), and cattle (Farm BH) farm. Based on the results of phylogenetic and K-means cluster analyses, the AiV-D strains formed three distinct host-specific lineages/clusters which indicate the widespread presence of different, host-specific AiV-D viruses in the examined ruminant farms in Hungary with no obvious signs of inter-species transmissions of these viruses between the investigated hosts. Based on these results, the members of species *Aichivirus D* are detected not just in caprine but for the first time in Europe in cattle and ovine as well.

The fourth picornavirus provisionally called capripivirus shows a shared relationship to sister clades of bopi- and erboviruses of subfamily *Caphthovirinae*; it has type-II IRES and NPGP-type 2A similar as bopi- and erboviruses (33, 47). On the other hand, based on the different phylogenetic positions of CapV in 3D^RdRp^ (more related to erboviruses) and P1 capsid trees (more related to bopiviruses), the separation of CapV from erbo- and bopiviruses in the K-means cluster analysis, the relatively low sequence identities to the closest relatives, and certain unique genome features like 3B^VPg^ duplication or distinct 3′UTR, CapV is different from the bopi- and erboviruses and could be the founding member of a novel genus, although based on the similar leaderless genome organization and the current sequence-based genus demarcation criteria (divergence of the orthologous proteins must exceed 66% of P1^cap^ and 64% of 2C^hel^, 3C^pro^, and 3D^pol^, https://www.picornastudygroup.com/definitions/genus_definition.htm), CapV could belong to genus *Bopivirus* as a founding member of a novel, but phylogenetically highly divergent species. Capripivirus was detectable by qPCR in several caprine and ovine samples originating from multiple different farms. In ovine, certain 3D^RdRp^ sequences were highly similar (98% aa identity, only 1/82 aa difference) to caprine CapV while others were relatively divergent (share only <85% nt identity) indicating a high sequence divergence of certain field strains (potentially different genotypes) of CapVs which could be endemic and widespread in Hungarian populations of investigated small ruminants with the possibility of inter-species transmission between caprine and ovine.

Based on the results of phylogenetic and genetic distance (p-dist) analyses, the caprine astrovirus strain goat/MAstV/KT-G5/2020-HUN most likely belongs to a recently proposed genotype species (“Caprine Astrovirus G5.1”) of genus *Mamastrovirus* together with other caprine and ovine AstVs from Switzerland and China (29 – 31). The goat/MAstV/KT-G5/2020-HUN shows the closest relationship to a CaAstV strain G5.1 which was previously identified as a caprine-ovine AstV recombinant strain (29). These data indicate the widespread presence of this type of CaAstV among caprine populations of different European countries. MAstVs were identifiable by qPCR in numerous ruminant samples originating from a single caprine and all investigated cattle and ovine farms and found to be the most common co-infecting virus. The identified MAstV RdRp sequences were separated into a mixed cluster of CaAstV G5.1-related AstVs from caprine and ovine hosts and two other clusters with multiple diverse AstV strains from cattle including a cluster which contains related AstV sequences from dogs, takin, and cattle (Fig. 11D). These data could indicate the potential cross-species transmission of certain cattle and caprine/ovine AstVs similar as suggested before (29, 48). There are multiple cattle farms (like Farm DR, TiV, or HB) where diverse AstV strains belonging to different clusters are present suggesting the simultaneous co-circulations of diverse AstVs in the investigated farms. Capsid sequences could be determined only from two cattle from the same farm and four additional caprine samples which originated from the same caprine farm (Farm KT) as the index CaAstV strain goat/MAstV/KT-G5/2020-HUN. These CaAstV capsid sequences are completely identical to each other which indicates the widespread presence of this MAstV type in the investigated caprine farm.

Prevalence of the investigated RNA viruses and as co-infection rates were considerably higher in less than 1-year-old animals than in adults which indicates the higher susceptibility for infections of these viruses of youngsters especially <2 month olds, where quadruple and quintuple infections were also observed. Co-infections of the RNA viruses were detected in all ruminants investigated, but much higher percentages were found in small ruminants (up to five co-infecting viruses in certain cases) than in cattle (only dual infections were found) most likely due to the different housing conditions (single caprine/ovine herds vs separate pens with cattle of the same age; young calves held in single pens).

In this study, a total of seven viruses belonging to four different viral families were described from diarrheic caprine which shows close phylogenetic relationships to certain human and ruminant livestock viruses suggesting the cross-species transmission capabilities between small ruminants and in the case of tusavirus and CRESS DNA virus a (reverse) zoonotic spread of these viruses. The high diversity of RNA viruses typically found as part of multiple co-infections mostly in young animals of caprine, ovine, and cattle could facilitate the recombination events between co-infecting viruses as could happened between caprine AiV-D and BooV where certain IRES stem loops could be exchanged. Caprine farming includes “large-scale industrial livestock farms” for mass production of meat and/or milk, but the goats which are mostly kept together with other livestock like ovine are frequently found in small backyard farms and petting zoos with close contact to humans which could hold the unknown threat to humans and companion animals as well.

## MATERIALS AND METHODS

### Background information on samples, animals, and farms

In this study, a total of 188 fecal samples were used, which were collected from mostly asymptomatic (only *n* = 11 caprine and *n* = 6 cattle were diarrheic, Table S3) ruminants like *Capra aegagrus hircus* (domestic caprine, *n* = 62), *Ovis aries* (domestic sheep, *n* = 32), and *Bos taurus* (domestic cattle, *n* = 94) from a total of 12 geographically distant farms (*n* = 2 ovine, *n* = 4 caprine, and *n* = 6 cattle) across Hungary, between 2008 and 2020 (Fig. 10). There is no available information about the previous medical history or the feeding methods of the investigated animals. Most of the investigated farms were “large-scale industrial livestock farms” in which ≈100 to >1,000 animals were held together, except caprine farms of Nagyhegy (≈10 animals) and Aranyosgadány (≈50 animals) and a cattle farm of Nyíregyháza (≈20 animals). All the ovine and caprine (except for Győrszentiván) farms, as well as a single cattle farm (Derecske), were extensive management-type farms where animals of different ages were released freely during the daytime and were housed together indoors at night (Table 2). In contrast, the other *n* = 5 cattle and a caprine farm of Győrszentiván were intensive management-type farms, where animals were always restricted to the closed indoor stockyards where the goats were kept together in multiple herds while the cattle were kept in separate pens; animals of similar age groups were kept together (Table 2). The index caprine farm of Győrszentiván (Farm KT) from which the samples for next-generation sequencing (NGS) were collected was a large-scale dairy goat farm with more than 200 mother goats, where the kids were separated from their mothers within a few weeks after birth and fed artificially by feeding bottles. The animals with apparent health problems which were mostly young, less than 1-year-old animals (e.g., gastroenteritis, restlessness, or nasal discharge), are separated from the flock but kept together in a distinct stockyard. Fecal samples were collected from the flooring underneath the animals into sterile tubes, transported in cooled containers, and kept at −20°C until processing. The sampled animals were classified into three approximate age groups (group I: <2 months old; group II: 2–12 months old; and group III: >12 months old) (Table 2; Table S3).

### Viral metagenomics, next-generation sequencing, and NGS data analyses

Details of sample processing, viral metagenomics, and NGS as well as detailed descriptions of the bioinformatics pipeline applied in this study can be found in our previous report (49). Briefly, a single-sample pool (1:1 ratio) was created from fecal samples of three less than 1-year-old caprine with the signs of gastroenteritis (KT-G4, KT-G5, and KT-FG3) which were collected on a farm from Győrszentiván (Farm KT). The unprotected nucleic acids from the filtered fecal sample pool were digested with a nuclease cocktail. After nucleic acid isolation and reverse transcription, DNA was amplified by random PCR. After library construction, the sample was run on a NovaSeq 6000 (Illumina, San Diego, USA) platform and the resulting sequence data were analyzed with an “*in-house*” developed bioinformatics pipeline using Kaiju v1.7.3, DIAMOND v2.1.6 aligner, and MEGAN6 v6.24.22 software with the NCBI RefSeq databases for initial virus identification (50 – 52). The filtered and quality-checked reads were *de novo* assembled, and the generated reads and contigs were mapped to reference sequences (Fig. S1) using Geneious Prime Ver. 2022.1.1 (Biomatters, New Zealand). The reference sequences were identified using BLAST analyses of the generated contigs.

### RT-PCR-based genome acquisition reactions

Total nucleic acids were extracted separately from 150 µL ~ 40(vol/vol%) fecal suspensions of KT-G4, KT-G5, and KT-FG3 diluted in sterile 0.1M phosphate-buffered saline (PBS) using Quick-RNA Viral Kit (Zymo Research, Irvine, USA) according to the manufacturer’s instructions but without DNAse treatment. For complete genome/coding sequence determinations, first individual sample(s) from the NGS sample pool which contains the virus(es) detected by NGS analyses were identified using virus-specific RT-PCR reactions. Then, for each virus which were present in multiple samples of the NGS sample pool, one of the positive samples was chosen for further genome determination RT-PCR reactions. To avoid the creation of chimeric sequences from the NGS reads of the pooled sample, the genome of a given virus was assembled from the sequences of overlapping PCR products determined by Sanger sequencing from a single sample (no reads and contigs were included to the final sequence) except the CRESS DNA virus which was present in a single sample of the pool (KT-G4); therefore, the mapped reads and contigs were also used for the assembly of the complete genome. The ID (KT-G4, KT-G5, or KT-FG3) of the chosen sample was part of the strain name of the given virus. For genome determination reactions, different RT-PCR techniques were used including terminal deoxynucleotidyl transferase (TdT, Thermo-Fisher, Waltham, USA) enzyme-based 5′ RACE (rapid amplification of cDNA ends) methods (53). The applied 5′ RACE protocols had a synthesis step of either poly cytosine, guanine, or adenine tracks with the use of the TdT. Generic oligonucleotide primers used for primer-walking-type genome acquisition reactions were designed based on the *de novo* assembled contigs and nucleotide (nt) alignments of the generated NGS reads to the selected reference sequences (Fig. S1). The conditions and reagents used in the genome acquisition RT-PCR reactions were the same as described previously (16).

The generated PCR products were sequenced directly in both directions using the BigDye Terminator v1.1 Cycle Sequencing Kit (Thermo Fisher, Waltham, USA) on an ABI 3500 Genetic Analyzer (Applied Biosystems, Hitachi, Tokyo, Japan).

### SYBRgreen-based qPCR and capsid determination reactions

For qPCR and capsid determination reactions, total RNA was extracted from fecal samples using TRI Reagent (MRC, USA) according to the manufacturer’s instructions.

For the screening qPCR reactions, 5 µL total RNA was converted to cDNA using random hexamer and Oligo dT primers (Thermo Fisher, Waltham, USA) and M-MLV-RT enzyme (Promega, Madison, USA) in a reverse transcription reaction (20 µL total volume) in a 96-well plate format (Fig. S4). The total of two RT plates were incubated at 22°C for 10 min followed by 42°C for 45 min. The obtained cDNA plates were used for further SYBRgreen-based qPCR and capsid determination PCR reactions.

For SYBRg-based qPCR reactions, Luna Universal qPCR Master Mix (New England Biolabs, Ipswich, USA) was used according to the manufacturer’s instructions. All SYBRg-qPCR reactions were supplemented with a melting curve analysis and run on a 96-well format in a CFX96 Touch System (Bio-Rad, Hercules, USA). Data analyses were made with a CFX-Maestro software ver. 4.1.

In the total of five different singleplex qPCR assays, separate virus group-specific primer pairs were used which were designed to amplify the conserved RdRp regions of the determined caprine RNA viruses and their closest relatives identified by BLAST searches (Table S, S2; Fig. S4). Based on the measured melting temperatures of the positive controls (study RNA virus strains with determined complete genomes), the results of subsequent agarose gel-electrophoretic separations (Fig. S4), and sequence analyses of qPCR products, samples with Ct < 38.0 and melting temperatures (Tm) ± 1.0°C of the Tm of positive control (in case of kobuviruses Tm ±2.0°C, due to the relatively high GC content) of the given assay were considered to be qPCR positive. To investigate the genotype variance of the strains detected with qPCR assays, PCR reactions were conducted on all the qPCR-positive samples using “*in-house*” designed generic primer pairs targeting various capsid regions (VP1 of picornaviruses and N-terminal capsid of astroviruses) of the studied virus groups. The multiple sets of generic primer pairs were designed to the alignment of determined caprine RNA viruses and their closest relatives identified by BLAST searches (Table S2; Fig. S4). The generated PCR products were sequenced in a similar way as described in the previous section of (*RT-)PCR-based genome acquisition reactions*.

### *In silico* sequence and phylogenetic analyses

The sequences related most closely to the sturdy strains were identified by NCBI’s BLAST search tools (54).

Multiple sequence alignments used for primer design, phylogenetic analyses, and sequence comparisons were generated on the online platforms of Multiple Sequence Comparison by Log-Expectation (MUSCLE, https://www.ebi.ac.uk/Tools/msa/muscle/), Clustal Omega (https://www.ebi.ac.uk/Tools/msa/clustalo/), or MAFFT (55) with default parameters. Possible proteolytic cleavage sites of the study viruses were predicted based on the individual aa alignments with the closest relatives which have known/predicted cleavage sites. The possible secondary RNA structures of the 5′UTRs of the study viruses were modelled using the Mfold software and visualized using VARNA and CorelDraw Graphics Suite v. 12. software (56, 57). GeneDoc software ver. 2.7 and Geneious Prime Ver. 2022.1.1 (Biomatters, New Zealand) were used for sequence assembly, as well as pairwise nucleotide (nt) and amino acid (aa) identity calculations. Nt and deduced aa sequence-based phylogenetic trees were constructed using either IQ-TREE webserver or MEGA software ver. 11.0 with the maximum-likelihood method and various models specified in the legends of relevant figures (58, 59). The K-means cluster analysis script was written under R/RStudio using msa, ape, seqinr, and factoextra libraries (59 – 63). Using the factoextra package, the clustering of the data was examined based on the Hopkins statistical method, determined the optimal number of clusters, and finally clustered the data. If the Hopkins statistic (64) is less than 0.5, the data are “uniformly distributed;” if the value is close to 1, the sequences “contain meaningful clusters.”

## Data Availability

The complete genome/coding sequences of study viruses can be found in GenBank under the accession numbers OQ758025-OQ758030 and OL692339.2. The additional *n* = 77 partial RdRp and capsid sequences determined in this study are available under accession numbers OQ758031-OQ758107.

## SUPPLEMENTAL MATERIAL

Supplemental material spectrum.02533-23-s0001.docx Tables S1 to S4 and Fig. S1 to S4.

ASM does not own the copyrights to Supplemental Material that may be linked to, or accessed through, an article. The authors have granted ASM a non-exclusive, world-wide license to publish the Supplemental Material files. Please contact the corresponding author directly for reuse.
