# Supplementary material for: The genomic and epidemiological investigations of enteric viruses of domestic caprine (Capra hircus) revealed the presence of multiple novel viruses related to known strains of humans and ruminant livestock species
Source: Microbiol Spectr. 2023 Oct 12;11(6):e02533-23. doi: 10.1128/spectrum.02533-23 (PMC10714811; doi:10.1128/spectrum.02533-23)
Supplement: Supplemental material — Tables S1 to S4 and Fig. S1 to S4. [file spectrum.02533-23-s0001.docx]

| **Assay name** | **Target viruses of the SYBRg-qPCR assays** | **Host** | **Classification** | **Acc. No.** |
| --- | --- | --- | --- | --- |
| **CapV** | **goat/CapV/KT-G5/2020-HUN** | caprine (*Capra aegagrus hircu*s) | “Capripivirus” | OQ758027 |
| **BooV-C** | Ovine PV England/2004/E1028-04 | ovine (*Ovis aries*) | *Boosepivirus C* | LR216006 |
|  | Ovine PV Wales/2017/S014177 |  |  | LR216007 |
|  | Ovine PV Wales/2017/S014175 |  |  | LR216008 |
|  | Ovine PV England/2010/S78-04-10-1 |  |  | LR216011 |
|  | Ovine PV England/2004/E1029-04 |  |  | LR216012 |
|  | **goat/BooV/KT-FG-3/2020-HUN** | caprine (*Capra aegagrus hircus*) |  | OQ758026 |
| **EV-G** | Ovine EV ovine/TB4-OEV/2009/HUN | ovine (*Ovis aries*) | *Enterovirus G* | JQ277724.1 |
|  | Ovine EV NX-DR26 |  |  | MN598038.1 |
|  | Ovine EV 2019-00927 |  |  | OV176440.1 |
|  | Enterovirus G 990/UK-NI |  |  | MG958646.1 |
|  | Enterovirus sp. 66-k141_25260 |  |  | MZ679284.1 |
|  | Enterovirus sp. 344R-k141_987796 |  |  | MZ679311.1 |
|  | Goat EV goat/JL14 | caprine (*Capra aegagrus hircus*) |  | KU297674.1 |
|  | Goat EV JL-LS34 |  |  | MN598033.1 |
|  | Goat EV JL-LS127 |  |  | MN598034.1 |
|  | Goat EV JL-LS165 |  |  | MN598035.1 |
|  | Goat EV JL-LS174 |  |  | MN598036.1 |
|  | Goat EV SD-S68 |  |  | MN598040.1 |
|  | Goat EV NMG-F37 |  |  | MN598041.1 |
|  | **goat/EV/KT-FG3/2020-HUN** |  |  | OQ758028 |
| **AiV-D** | cattle/Kagoshima-2-24-KoV/2015/JPN | cattle (*Bos taurus*) | *Aichivirus D* | LC055960.1 |
|  | SKoV-China/SWUN/AB18/2019 | ovine (*Ovis aries*) |  | MW296158.1 |
|  | **goat/KoV/KT-G4/2020-HUN** | caprine (*Capra aegagrus hircus*) |  | OQ758029 |
| **MAstV** | S5.1 | ovine (*Ovis aries*) | *"Caprine Astrovirus G51." (Mamastrovirus)* | MK404647 |
|  | S6.1 |  |  | MZ005893 |
|  | G5.1 | caprine (*Capra aegagrus hircus*) |  | MK404648 |
|  | China/SWUN/F4/2019 |  |  | MK404649 |
|  | **goat/MAstV/KT-G5/2020-HUN** |  |  | OQ758025 |

**Table S1:** Names, host, classification and accession numbers (Acc. No.) of target viruses used for the primer design of specific SYBRg-qPCR assays of this study. The sequences were selected based on the results of BLAST searches of the study strains with determined complete genomes (in **bold**). Capripivirus (CapV), Boosepivirus C (BooV-C), Enterovirus G (EV-G), Aichivirus D (AiV-D) and goat/MAstV/KT-G5/2020-HUN-related astroviruses (MAstV).

| **Target virus** | **Assay type** | **Primer name** | **Primer sequence (5' - 3')** | **Product length (bp)** |
| --- | --- | --- | --- | --- |
| BooV-C | SYBRg-qPCR | gOviPV-SYBR-scr-F | TTT TCT AGT GTT AAG TGT AAT TT | 288 |
|  |  | gOviPV-SYBR-scr-R | CAC TGA TGT TAT RTG ACC TTG |  |
|  | RT-PCR genotyping | gOviPV-CAP-Fgen | CTG TCT ATA CAR GAG GGT GAT | 908 |
|  |  | gOviPV-CAP-Rgen | AAA GGT GGG GGA TTA TTC CT |  |
| EV-G | SYBRg-qPCR | gEV-SYBR-scr-F | CCC CGC CGA ATT GGC CAA | 270 |
|  |  | gEV-SYBR-scr-R | TAC TCC TTC CTG CCA TTA TGC CA |  |
|  | RT-PCR genotyping | gEV-CAP-Fgen | ATG TTT ACT GGG ACA TTY ATG AC | 1325 |
|  |  | gEV-CAP-Rgen | TCC CAC TCA AGA TTA TCC CA |  |
| AiV-D | SYBRg-qPCR | gKV-SYBR-scr-F | ATC ATC GAA CAC TAC CAG GC | 340 |
|  |  | gKV-SYBR-scr-R | ACA GCC ACG TTG TTG CAC CAG |  |
|  | RT-PCR genotyping | gKV-CAP-Fgen | CTT GTC GAT TCC CTA CGC CAA CCC | 372 |
|  |  | gKV-CAP-Rgen | TCA YKG TCY TGG CAC TGG CGC CG |  |
| CapV | SYBRg-qPCR | gCapV-SYBR-scr-F | GAC TAC TCT GAA CAY GTG TTT CA | 290 |
|  |  | gCapV-SYBR-scr-R | CTT TGG AAT GGG AAG CAT CAA |  |
|  | RT-PCR genotyping | gCapV-CAP-Fgen | ACA TAT GTG ATC TGG GAC ATT GG | 875 |
|  |  | gCapV-CAP-Rgen | GGG ACT GTG AAC TGC AAC CC |  |
| MAstV | SYBRg-qPCR | gAstV-SYBR-scr-F | GAT GTG TTT GGG ATG TGG GT | 329 |
|  |  | gAstV-SYBR-scr-R | TCC TCC CCT CCA AAG ACG ATC CAG |  |
|  | RT-PCR genotyping | gAstV-CAP-Fgen | TTC CAG AAG ATT ACG ACA ACA CTC | 948 |
|  |  | gAstV-CAP-Rgen | ATC TCC AAT GCC TGT GTT GCC |  |

**Table S2:** List and features of oligonucleotide primers used for epidemiological investigations (SYBRg-qPCR) and capsid determination reactions (RT-PCR genotyping). Boosepivirus C (BooV-C), Enterovirus G (EV-G), Aichivirus D (AiV-D), Capripivirus (CapV) and goat/MAstV/KT-G5/2020-HUN-related astroviruses (MAstV).

| **Farm ID** | **Sample ID** | **Host** | **Age groups** | **CapV** | **BooV-C** | **EV-G** | **AiV-D** | **MAstV** |
| --- | --- | --- | --- | --- | --- | --- | --- | --- |
| Farm K | K-1 | caprine | II | - | - | - | - | - |
| Farm K | K-2 | caprine | II | - | - | pos. | - | - |
| Farm K | K-3 | caprine | II | - | - | - | - | - |
| Farm K | K-4 | caprine | II | - | - | - | - | - |
| Farm K | K-5 | caprine | II | - | - | - | - | - |
| Farm K | K-6 | caprine | II | - | - | - | - | - |
| Farm K | K-7 | caprine | II | - | - | pos. | - | - |
| Farm K | K-8 | caprine | II | - | - | - | - | - |
| Farm K | K-9 | caprine | II | - | - | pos. | - | - |
| Farm K | K-10 | caprine | II | - | - | - | - | - |
| Farm K | K-11 | caprine | II | - | - | - | - | - |
| Farm K | K-12 | caprine | II | - | - | - | - | - |
| Farm AGK | AGK-1 | caprine | III | - | - | - | - | - |
| Farm AGK | AGK-2 | caprine | III | - | - | - | - | - |
| Farm AGK | AGK-3 | caprine | III | - | - | - | - | - |
| Farm AGK | AGK-4 | caprine | III | - | - | - | - | - |
| Farm AGK | AGK-5 | caprine | III | - | - | - | - | - |
| Farm AGK | AGK-6 | caprine | III | - | - | pos. | pos. | - |
| Farm AGK | AGK-7 | caprine | III | - | - | - | - | - |
| Farm AGK | AGK-8 | caprine | III | - | - | - | - | - |
| Farm AGK | AGK-9 | caprine | II | - | - | - | - | - |
| Farm AGK | AGK-10 | caprine | II | - | - | - | - | - |
| Farm AGK | AGK-11 | caprine | II | pos. | - | pos. | - | - |
| Farm AGK | AGK-12 | caprine | II | - | - | - | - | - |
| Farm AGK | AGK-13 | caprine | II | - | - | - | - | - |
| Farm AGK | AGK-14 | caprine | II | - | - | - | - | - |
| Farm AGK | AGK-15 | caprine | II | - | - | - | - | - |
| Farm AGK | AGK-16 | caprine | II | - | - | - | - | - |
| Farm NH | NH-1 | caprine | III | - | - | pos. | - | - |
| Farm NH | NH-2 | caprine | III | - | - | - | - | - |
| Farm NH | NH-3 | caprine | III | - | - | pos. | - | - |
| Farm NH | NH-4 | caprine | III | - | - | - | - | - |
| Farm NH | NH-5 | caprine | III | - | - | - | - | - |
| Farm KT | KT-FI-1 | caprine | III | - | - | pos. | pos. | pos. |
| Farm KT | KT-FI-2 | caprine | III | - | - | - | pos. | pos. |
| Farm KT | KT-FI-3 | caprine | III | - | - | - | pos. | - |
| Farm KT | KT-FI-4 | caprine | III | pos. | - | - | pos. | - |
| Farm KT | KT-FI-5 | caprine | III | - | - | - | - | - |
| Farm KT | KT-FII-1 | caprine | III | - | - | - | - | pos. |
| Farm KT | KT-FII-2 | caprine | III | - | pos. | pos. | pos. | - |
| Farm KT | KT-FII-3 | caprine | III | - | - | pos. | - | - |
| Farm KT | KT-FII-4 | caprine | III | - | pos. | pos. | - | - |
| Farm KT | KT-FII-5 | caprine | III | - | - | - | - | pos. |
| Farm KT | KT-G1* | caprine | II | pos. | - | - | pos. | pos. |
| Farm KT | KT-G2* | caprine | II | pos. | pos. | - | - | pos. |
| Farm KT | KT-G3* | caprine | II | pos. | pos. | - | - | pos. |
| Farm KT | **KT-G4*** | caprine | II | pos. | - | - | pos. | pos. |
| Farm KT | **KT-G5*** | caprine | II | pos. | pos. | - | - | pos. |
| Farm KT | KT-G6 | caprine | II | - | - | - | pos. | pos. |
| Farm KT | KT-G7 | caprine | II | - | - | - | - | pos. |
| Farm KT | KT-G8 | caprine | II | pos. | - | - | - | - |
| Farm KT | KT-G9 | caprine | II | pos. | - | - | pos. | pos. |
| Farm KT | KT-G10 | caprine | II | pos. | - | - | pos. | - |
| Farm KT | KT-FG2* | caprine | I | pos. | pos. | pos. | pos. | pos. |
| Farm KT | **KT-FG3*** | caprine | I | pos. | pos. | pos. | pos. | pos. |
| Farm KT | KT-FG4 | caprine | I | - | - | pos. | - | pos. |
| Farm KT | KT-FG5* | caprine | I | - | pos. | pos. | pos. | pos. |
| Farm KT | KT-FG6 | caprine | I | - | pos. | pos. | - | pos. |
| Farm KT | KT-FG7* | caprine | I | - | - | pos. | pos. | pos. |
| Farm KT | KT-FG8 | caprine | I | pos. | - | pos. | - | - |
| Farm KT | KT-FG9* | caprine | I | - | - | pos. | - | pos. |
| Farm KT | KT-FG10* | caprine | I | pos. | pos. | pos. | - | pos. |
| Farm TB | TB-1 | ovine | I | - | - | pos. | pos. | pos. |
| Farm TB | TB-2 | ovine | I | - | - | - | pos. | pos. |
| Farm TB | TB-3 | ovine | I | - | - | - | - | pos. |
| Farm TB | TB-4 | ovine | I | - | - | pos. | - | pos. |
| Farm TB | TB-5 | ovine | I | pos. | - | pos. | pos. | pos. |
| Farm TB | TB-6 | ovine | I | - | - | - | - | pos. |
| Farm TB | TB-7 | ovine | I | - | - | - | - | - |
| Farm TB | TB-8 | ovine | I | - | - | pos. | - | pos. |
| Farm TB | TB-9 | ovine | I | - | - | pos. | pos. | - |
| Farm TB | TB-10 | ovine | I | - | - | pos. | pos. | - |
| Farm TB | TB-11 | ovine | I | - | - | pos. | pos. | - |
| Farm TB | TB-12 | ovine | I | - | - | - | - | - |
| Farm TB | TB-13 | ovine | I | - | - | pos. | - | - |
| Farm TB | TB-14 | ovine | I | - | - | pos. | pos. | pos. |
| Farm TB | TB-15 | ovine | I | - | - | pos. | pos. | - |
| Farm TB | TB-16 | ovine | I | - | - | pos. | pos. | - |
| Farm HBSz | HBSZ-GI-1 | ovine | II | - | - | pos. | pos. | pos. |
| Farm HBSz | HBSZ-GI-2 | ovine | II | pos. | - | pos. | pos. | - |
| Farm HBSz | HBSZ-GI-3 | ovine | II | pos. | - | pos. | - | pos. |
| Farm HBSz | HBSZ-GI-4 | ovine | II | pos. | - | pos. | - | pos. |
| Farm HBSz | HBSZ-GI-5 | ovine | II | pos. | - | pos. | - | pos. |
| Farm HBSz | HBSZ-GII-1 | ovine | I | - | - | pos. | pos. | pos. |
| Farm HBSz | HBSZ-GII-2 | ovine | I | - | - | pos. | pos. | pos. |
| Farm HBSz | HBSZ-GII-3 | ovine | I | - | - | pos. | - | pos. |
| Farm HBSz | HBSZ-GII-4 | ovine | I | - | - | pos. | - | - |
| Farm HBSz | HBSZ-GII-5 | ovine | I | - | - | pos. | - | - |
| Farm HBSz | HBSZ-GIII-1 | ovine | I | - | - | - | pos. | - |
| Farm HBSz | HBSZ-GIII-2 | ovine | I | - | - | pos. | pos. | pos. |
| Farm HBSz | HBSZ-GIII-3 | ovine | I | pos. | - | pos. | pos. | - |
| Farm HBSz | HBSZ-GIV-1 | ovine | III | - | - | pos. | pos. | pos. |
| Farm HBSz | HBSZ-GIV-2 | ovine | III | - | - | pos. | pos. | - |
| Farm HBSz | HBSZ-TL-1 | ovine | III | - | - | - | - | - |
| Farm HB | HB-7369 | cattle | I | - | - | - | pos. | pos. |
| Farm HB | HB-7660 | cattle | I | - | - | - | - | - |
| Farm HB | HB-7373 | cattle | I | - | - | - | pos. | pos. |
| Farm HB | HB-7431* | cattle | I | - | - | - | - | - |
| Farm HB | HB-7398* | cattle | I | - | - | - | - | pos. |
| Farm HB | HB-7693 | cattle | I | - | - | - | - | - |
| Farm HB | HB-7433 | cattle | I | - | - | - | - | pos. |
| Farm HB | HB-7248 | cattle | I | - | - | - | - | - |
| Farm HB | HB-7615* | cattle | I | - | - | - | - | - |
| Farm HB | HB-7315 | cattle | I | - | - | - | pos. | pos. |
| Farm HB | HB-7657* | cattle | I | - | - | - | - | pos. |
| Farm HB | HB-7500 | cattle | I | - | - | - | - | - |
| Farm HB | HB-7673* | cattle | I | - | - | - | - | - |
| Farm HB | HB-E-1 | cattle | III | - | - | - | - | pos. |
| Farm HB | HB-7627 | cattle | I | - | - | - | - | pos. |
| Farm HB | HB-7751* | cattle | I | - | - | - | - | - |
| Farm HB | HB-8066 | cattle | I | - | - | - | - | pos. |
| Farm HB | HB-7663 | cattle | I | - | - | - | - | - |
| Farm HB | HB-7394 | cattle | I | - | - | - | - | pos. |
| Farm HB | HB-P1 | cattle | III | - | - | - | pos. | pos. |
| Farm NyH | NYH-3175 | cattle | I | - | - | - | - | pos. |
| Farm NyH | NYH-3254 | cattle | I | - | - | - | - | - |
| Farm NyH | NYH-3257 | cattle | I | - | - | - | pos. | pos. |
| Farm NyH | NYH-3259 | cattle | I | - | - | - | - | pos. |
| Farm NyH | NYH-3256 | cattle | I | - | - | - | - | pos. |
| Farm NyH | NYH-GI-1 | cattle | II | - | - | - | pos. | - |
| Farm NyH | NYH-GI-2 | cattle | II | - | - | - | pos. | pos. |
| Farm NyH | NYH-GII-1 | cattle | II | - | - | - | pos. | - |
| Farm NyH | NYH-GII-2 | cattle | II | - | - | - | pos. | pos. |
| Farm NyH | NYH-GIII-1 | cattle | III | - | - | - | pos. | pos. |
| Farm NyH | NYH-GIII-2 | cattle | III | - | - | - | pos. | - |
| Farm DR | DR-1 | cattle | III | - | - | - | pos. | pos. |
| Farm DR | DR-2 | cattle | III | - | - | - | pos. | - |
| Farm DR | DR-3 | cattle | III | - | - | - | - | pos. |
| Farm DR | DR-4 | cattle | III | - | - | - | - | pos. |
| Farm DR | DR-5 | cattle | III | - | - | - | pos. | - |
| Farm DR | DR-6 | cattle | III | - | - | - | - | pos. |
| Farm DR | DR-7 | cattle | III | - | - | - | pos. | - |
| Farm DR | DR-8 | cattle | III | - | - | - | pos. | - |
| Farm DR | DR-9 | cattle | III | - | - | - | pos. | - |
| Farm DR | DR-10 | cattle | III | - | - | - | pos. | pos. |
| Farm DR | DR-11 | cattle | III | - | - | - | - | - |
| Farm DR | DR-2 | cattle | I | - | - | - | pos. | pos. |
| Farm DR | DR-3 | cattle | I | - | - | - | pos. | pos. |
| Farm DR | DR-4 | cattle | II | - | - | - | pos. | - |
| Farm TiV | TiV-001 | cattle | I | - | - | - | - | - |
| Farm TiV | TiV-002 | cattle | I | - | - | - | - | - |
| Farm TiV | TiV-003 | cattle | I | - | - | - | - | - |
| Farm TiV | TiV-004 | cattle | I | - | - | - | - | - |
| Farm TiV | TiV-005 | cattle | I | - | - | - | pos. | pos. |
| Farm TiV | TiV-GI-1 | cattle | II | - | - | - | pos. | pos. |
| Farm TiV | TiV-GI-2 | cattle | II | - | - | - | pos. | pos. |
| Farm TiV | TiV-GI-3 | cattle | II | - | - | - | pos. | pos. |
| Farm TiV | TiV-GII-1 | cattle | II | - | - | - | - | - |
| Farm TiV | TiV-GII-2 | cattle | II | - | - | - | pos. | pos. |
| Farm TiV | TiV-GIII-1 | cattle | II | - | - | - | pos. | pos. |
| Farm TiV | TiV-GIII-2 | cattle | II | - | - | - | pos. | pos. |
| Farm TiV | TiV-F1 | cattle | III | - | - | - | - | - |
| Farm TiV | TiV-007 | cattle | I | - | - | - | - | pos. |
| Farm TiV | TiV-6379 | cattle | I | - | - | - | - | - |
| Farm TiV | TiV-0577 | cattle | I | - | - | - | - | pos. |
| Farm BH | BH-0001 | cattle | I | - | - | - | - | pos. |
| Farm BH | BH-9812 | cattle | I | - | - | - | - | pos. |
| Farm BH | BH-9852 | cattle | I | - | - | - | pos. | - |
| Farm BH | BH-9854 | cattle | I | - | - | - | - | pos. |
| Farm BH | BH-9858 | cattle | I | - | - | - | - | - |
| Farm BH | BH-9861 | cattle | I | - | - | - | pos. | - |
| Farm BH | BH-9865 | cattle | I | - | - | - | - | - |
| Farm BH | BH-9878 | cattle | I | - | - | - | pos. | pos. |
| Farm BH | BH-9901 | cattle | I | - | - | - | - | pos. |
| Farm BH | BH-9955 | cattle | I | - | - | - | - | - |
| Farm BH | BH-9963 | cattle | I | - | - | - | - | pos. |
| Farm BH | BH-9971 | cattle | I | - | - | - | - | - |
| Farm BH | BH-9974 | cattle | I | - | - | - | - | pos. |
| Farm BH | BH-9989 | cattle | I | - | - | - | - | pos. |
| Farm BH | BH-9993 | cattle | I | - | - | - | - | pos. |
| Farm BH | BH-9994 | cattle | I | - | - | - | - | pos. |
| Farm TV | TV-9682 | cattle | I | - | - | - | - | pos. |
| Farm TV | TV-9686 | cattle | I | - | - | - | - | - |
| Farm TV | TV-9703 | cattle | I | - | - | - | pos. | pos. |
| Farm TV | TV-9728 | cattle | I | - | - | - | - | - |
| Farm TV | TV-9733 | cattle | I | - | - | - | - | - |
| Farm TV | TV-9736 | cattle | I | - | - | - | - | pos. |
| Farm TV | TV-9738 | cattle | I | - | - | - | pos. | pos. |
| Farm TV | TV-9743 | cattle | I | - | - | - | - | pos. |
| Farm TV | TV-9746 | cattle | I | - | - | - | - | pos. |
| Farm TV | TV-9760 | cattle | I | - | - | - | - | pos. |
| Farm TV | TV-9764 | cattle | I | - | - | - | - | - |
| Farm TV | TV-9772 | cattle | I | - | - | - | pos. | pos. |
| Farm TV | TV-9774 | cattle | I | - | - | - | - | pos. |
| Farm TV | TV-9779 | cattle | I | - | - | - | - | pos. |
| Farm TV | TV-9785 | cattle | I | - | - | - | - | pos. |
| Farm TV | TV-9787 | cattle | I | - | - | - | - | pos. |
| Farm TV | TV-9789 | cattle | I | - | - | - | - | pos. |

**Table S3**: Summarized results of SYBRg-qPCR assays of Boosepivirus C (BooV-C), Enterovirus G (EV-G), Aichivirus D (AiV-D), Capripivirus (CapV) and goat/MAstV/KT-G4/2020-HUN-related astroviruses (MAstV) with farm locations, sample IDs and age groups (group I: < 2-month-old, group II: 2–12-month-old, group III: >12-month-old). -: qPCR negative, pos.: qPCR positive (Ct <38.0 and melting temperatures (Tm) ±1.0/2.0^o^C of the Tm of positive control, contains a single band with the expected size, see Fig S4 for details). Co-infections are colour-coded: yellow: dual, green: triple, blue: quadruple and red: quintuple. * Animals showed the signs of diarrhoea. All other animals were apparently healthy.

| **Virus group/Assay name** | **Sequence ID** | **Strain name** | **Accession No.** | **Length (nt)** | **Host** | **Best hit (BLASTn) name** | **Best hit - Classification** | **Best hit - Host** | **Best hit - Acc. No.** | **nt identity (%)** |
| --- | --- | --- | --- | --- | --- | --- | --- | --- | --- | --- |
| MAstV | **KT-G5** | goat/MAstV/KT-G5/2020-HUN | OQ758025 | 286 | caprine | Caprine AstV G5.1 | *Mamastrovirus* | caprine | MK404647.1 | 88.42 |
|  | KT-G2 | goat/MAstV/KT-G2/2020-HUN | OQ758031 | 286 | caprine | Caprine AstV G5.1 | *Mamastrovirus* | caprine | MK404647.1 | 88.42 |
|  | HBSz-GI-3 | ovine/MAstV/HBSz-GI-3/2020-HUN | OQ758032 | 286 | ovine | Caprine AstV G5.1 | *Mamastrovirus* | caprine | MK404647.1 | 92.66 |
|  | HBSz-GI-4 | ovine/MAstV/HBSz-GI-4/2020-HUN | OQ758033 | 286 | ovine | Caprine AstV G5.1 | *Mamastrovirus* | caprine | MK404647.1 | 91.58 |
|  | TB2 | ovine/MAstV/TB2/2010-HUN | OQ758034 | 286 | ovine | Caprine AstV G5.1 | *Mamastrovirus* | caprine | MK404647.1 | 87.37 |
|  | TB5 | ovine/MAstV/TB5/2010-HUN | OQ758035 | 286 | ovine | Caprine AstV G5.1 | *Mamastrovirus* | caprine | MK404647.1 | 94.74 |
|  | BH-9854 | bovine/MAstV/BH-9854/2019-HUN | OQ758036 | 286 | cattle | BoAstV/JPN/Kagoshima2-52/2015 | *Mamastrovirus* | cattle | LC047801.1 | 92.28 |
|  | BH-9963 | bovine/MAstV/BH-9963/2019-HUN | OQ758037 | 286 | cattle | BoAstV/JPN/Hokkaido12-7/2009 | *Mamastrovirus* | cattle | LC047791.1 | 92.63 |
|  | DR-2 | bovine/MAstV/DR-2/2020-HUN | OQ758038 | 286 | cattle | BoAstv/CHN/HLJ-2/2019 | *Mamastrovirus* | cattle | MW373714 | 92.63 |
|  | DR-3 | bovine/MAstV/DR-3/2020-HUN | OQ758039 | 286 | cattle | BoAstV/JPN/Kagoshima1-2/2014 | *Mamastrovirus* | cattle | LC047795.1 | 93.33 |
|  | HB-7394 | bovine/MAstV/HB-7394/2020-HUN | OQ758040 | 286 | cattle | BoAstV/JPN/Kagoshima2-52/2015 | *Mamastrovirus* | cattle | LC047801.1 | 93.01 |
|  | HB-7398 | bovine/MAstV/HB-7398/2020-HUN | OQ758041 | 286 | cattle | AstroviridaeDogfe316C1 | *Mamastrovirus* | dog | OQ198051 | 91.63 |
|  | HB-8066 | bovine/MAstV/HB-8066/2020-HUN | OQ758042 | 286 | cattle | BoAstV66/2021/CHN | *Mamastrovirus* | cattle | ON682301.1 | 93.68 |
|  | NYH-3256 | bovine/MAstV/NYH-3256/2020-HUN | OQ758043 | 286 | cattle | AstroviridaeDogfe316C1 | *Mamastrovirus* | dog | OQ198051.1 | 92.31 |
|  | NYH-3259 | bovine/MAstV/NYH-3259/2020-HUN | OQ758044 | 286 | cattle | AstroviridaeDogfe316C1 | *Mamastrovirus* | dog | OQ198051.1 | 91.99 |
|  | TiV-0577 | bovine/MAstV/TiV-0577/2020-HUN | OQ758045 | 286 | cattle | BoAstv/CHN/Hebei-1/2019 | *Mamastrovirus* | cattle | MW373712.1 | 90.53 |
|  | TiV-GII-2 | bovine/MAstV/TiV-GII-2/2020-HUN | OQ758046 | 286 | cattle | BoAstV40/2021/CHN | *Mamastrovirus* | cattle | MW373712.1 | 93.33 |
|  | TV-9736 | bovine/MAstV/TV-9736/2019-HUN | OQ758047 | 286 | cattle | AstroviridaeDogfe316C1 | *Mamastrovirus* | dog | OQ198051.1 | 93.33 |
|  | TV-9738 | bovine/MAstV/TV-9738/2019-HUN | OQ758048 | 286 | cattle | AstroviridaeDogfe316C1 | *Mamastrovirus* | dog | OQ198051.1 | 93.33 |
| EV-G | **KT-FG-3** | goat/EV/KT-FG3/2020-HUN | OQ758028 | 229 | caprine | NX-DR26 | *Enterovirus G* | ovine | MN598038.1 | 87.34 |
|  | KT-FG-6 | goat/EV/KT-FG6/2020-HUN | OQ758064 | 229 | caprine | JL-LS127 | *Enterovirus G* | caprine | MN598034.1 | 90.79 |
|  | NH3 | goat/EV/NH3/2020-HUN | OQ758065 | 229 | caprine | JL-LS127 | *Enterovirus G* | caprine | MN598034.1 | 90.39 |
|  | NH1 | goat/EV/NH1/2020-HUN | OQ758066 | 229 | caprine | JL-LS127 | *Enterovirus G* | caprine | MN598034.1 | 90.39 |
|  | AGK11 | goat/EV/AGK11/2020-HUN | OQ758067 | 229 | caprine | JL-LS165 | *Enterovirus G* | caprine | MN598035.1 | 89.91 |
|  | AGK6 | goat/EV/AGK6/2020-HUN | OQ758068 | 229 | caprine | JL-LS165 | *Enterovirus G* | caprine | MN598035.1 | 89.91 |
|  | K2 | goat/EV/K2/2008-HUN | OQ758069 | 229 | caprine | TB4-OEV/2009/HUN | *Enterovirus G* | ovine | JQ277724.1 | 93.01 |
|  | K9 | goat/EV/K9/2008-HUN | OQ758070 | 229 | caprine | TB4-OEV/2009/HUN | *Enterovirus G* | ovine | JQ277724.1 | 93.01 |
|  | HBSz-GI-3 | ovine/EV/HBSz-GI-3/2020-HUN | OQ758071 | 229 | ovine | JL-LS174 | *Enterovirus G* | caprine | MN598036.1 | 90.39 |
|  | HBSz-GIV-1 | ovine/EV/HBSz-GIV-1/2020-HUN | OQ758072 | 229 | ovine | THAI/G2078 | *Enterovirus G* | caprine | MG583838.1 | 90.83 |
|  | TB1 | ovine/EV/TB1/2010-HUN | OQ758073 | 229 | ovine | Goat-EV/THAI/G2111 | *Enterovirus G* | caprine | MG583841.1 | 94.01 |
|  | TB10 | ovine/EV/TB10/2010-HUN | OQ758074 | 229 | ovine | TB4-OEV/2009/HUN | *Enterovirus G* | ovine | JQ277724.1 | 92.58 |
| AiV-D | **KT-G-4** | goat/KoV/KT-G4/2020-HUN | OQ758029 | 300 | caprine | BKV5/2021/CHN | *Aichivirus D* | cattle | ON730709.1 | 90.57 |
|  | KT-FI-1 | goat/KoV/KT-FI-1/2020-HUN | OQ758080 | 300 | caprine | BKV5/2021/CHN | *Aichivirus D* | cattle | ON730709.1 | 89.97 |
|  | KT-G1 | goat/KoV/KT-G1/2020-HUN | OQ758081 | 300 | caprine | BKV5/2021/CHN | *Aichivirus D* | cattle | ON730709.1 | 89.97 |
|  | AGK6 | goat/KoV/AGK6/2020-HUN | OQ758082 | 300 | caprine | 106-k141_523010 | *Aichivirus D* | cattle | MZ679297.1 | 90.30 |
|  | TB5 | ovine/KoV/TB5/2010-HUN | OQ758083 | 300 | ovine | SKoV-China/SWUN/AB18/2019 | *Aichivirus D* | ovine | MW296158.1 | 88.33 |
|  | TB16 | ovine/KoV/TB16/2010-HUN | OQ758084 | 300 | ovine | SKoV-China/SWUN/AB18/2019 | *Aichivirus D* | ovine | MW296158.1 | 86.33 |
|  | HBSz-GI-2 | ovine/KoV/HBSz-GI-2/2020-HUN | OQ758085 | 300 | ovine | SKoV-China/SWUN/AB18/2019 | *Aichivirus D* | ovine | MW296158.1 | 85.95 |
|  | HBSz-GIII-1 | ovine/KoV/HBSz-GIII-1/2020-HUN | OQ758086 | 300 | ovine | SKoV-China/SWUN/AB18/2019 | *Aichivirus D* | ovine | MW296158.1 | 86.96 |
|  | BH-9852 | bovine/KoV/BH-9852/2019-HUN | OQ758087 | 300 | cattle | Kagoshima-2-24-KoV/2015/JPN | *Aichivirus D* | cattle | LC055960.1 | 95.29 |
|  | BH-9878 | bovine/KoV/BH-9878/2019-HUN | OQ758088 | 300 | cattle | Kagoshima-2-24-KoV/2015/JPN | *Aichivirus D* | cattle | LC055960.1 | 95.65 |
|  | TV-9772 | bovine/KoV/TV-9772/2019-HUN | OQ758089 | 300 | cattle | 106-k141_523010 | *Aichivirus D* | cattle | MZ679297.1 | 95.65 |
|  | TV-9738 | bovine/KoV/TV-9738/2019-HUN | OQ758090 | 300 | cattle | 106-k141_523010 | *Aichivirus D* | cattle | MZ679297.1 | 94.31 |
|  | TiV-GIII-1 | bovine/KoV/TiV-GIII-1/2020-HUN | OQ758091 | 300 | cattle | 106-k141_523010 | *Aichivirus D* | cattle | MZ679297.1 | 95.67 |
|  | TiV-005 | bovine/KoV/TiV-005/2020-HUN | OQ758092 | 300 | cattle | 106-k141_523010 | *Aichivirus D* | cattle | MZ679297.1 | 94.33 |
|  | HB-7315 | bovine/KoV/HB-7315/2020-HUN | OQ758093 | 300 | cattle | Kagoshima-2-24-KoV/2015/JPN | *Aichivirus D* | cattle | LC055960.1 | 95.67 |
|  | HB-P1 | bovine/KoV/HB-P1/2020-HUN | OQ758094 | 300 | cattle | Kagoshima-2-24-KoV/2015/JPN | *Aichivirus D* | cattle | LC055960.1 | 96.67 |
|  | NYH-GIII-2 | bovine/KoV/NYH-GIII-2/2020-HUN | OQ758095 | 300 | cattle | Kagoshima-2-24-KoV/2015/JPN | *Aichivirus D* | cattle | LC055960.1 | 95.00 |
|  | NYH-GI-1 | bovine/KoV/NYH-GI-1/2020-HUN | OQ758096 | 300 | cattle | Kagoshima-2-24-KoV/2015/JPN | *Aichivirus D* | cattle | LC055960.1 | 96.00 |
|  | DR-1 | bovine/KoV/DR-1/2020-HUN | OQ758097 | 300 | cattle | Kagoshima-2-24-KoV/2015/JPN | *Aichivirus D* | cattle | LC055960.1 | 95.33 |
|  | DR-8 | bovine/KoV/DR-8/2020-HUN | OQ758098 | 300 | cattle | 106-k141_523010 | *Aichivirus D* | cattle | MZ679297.1 | 97.33 |
| CapV | **KT-G5** | goat/CapV/KT-G5/2020-HUN | OQ758027 | 248 | caprine | Erbovirus A strain 421 | *Erbovirus A* | horse | KX260140.1 | 66.67 |
|  | KT-G3 | goat/CapV/KT-G3/2020-HUN | OQ758058 | 248 | caprine | Erbovirus A strain 421 | *Erbovirus A* | horse | KX260140.1 | 66.67 |
|  | HBsz-GI-4 | ovine/CapV/HBsz-GI-4/2020-HUN | OQ758059 | 248 | ovine | Equine rhinitis B virus 2 strain 1228 | *Erbovirus A* | horse | KX260141.1 | 67.46 |
|  | HBSz-GI-2 | ovine/CapV/HBsz-GI-2/2020-HUN | OQ758060 | 248 | ovine | Equine rhinitis B virus 2 strain 1228 | *Erbovirus A* | horse | KX260141.1 | 67.46 |
|  | TB5 | ovine/CapV/TB5/2010-HUN | OQ758061 | 248 | ovine | Equine rhinitis B virus 2 strain 1228 | *Erbovirus A* | horse | KX260141.1 | 63.59 |
| BooV-C | **KT-FG-3** | goat/BooV/KT-FG-3/2020-HUN | OQ758026 | 244 | caprine | Ovine PV England/2010/S78-04-10-1 | *Boosepivirus C* | ovine | LR216011.1 | 74.68 |
|  | KT-G3 | goat/BooV/KT-G3/2020-HUN | OQ758055 | 244 | caprine | Ovine PV England/2004/E1029-04 | *Boosepivirus C* | ovine | LR216012.1 | 73.19 |
|  | KT-FG-5 | goat/BooV/KT-FG-5/2020-HUN | OQ758056 | 244 | caprine | Ovine PV England/2010/S78-04-10-1 | *Boosepivirus C* | ovine | LR216011.1 | 74.68 |

**Table S4**: Features of partial RNA-dependent RNA polymerase (RdRp) sequences determined from selected SYBRg-qPCR positive samples of this study and most similar sequences identified by BLAST similarity search. Sequence IDs are the same as found in Fig. 12. Capripivirus (CapV), Boosepivirus C (BooV-C), Enterovirus G (EV-G), Aichivirus D (AiV-D) and goat/MAstV/KT-G5/2020-HUN-related astroviruses (MAstV)


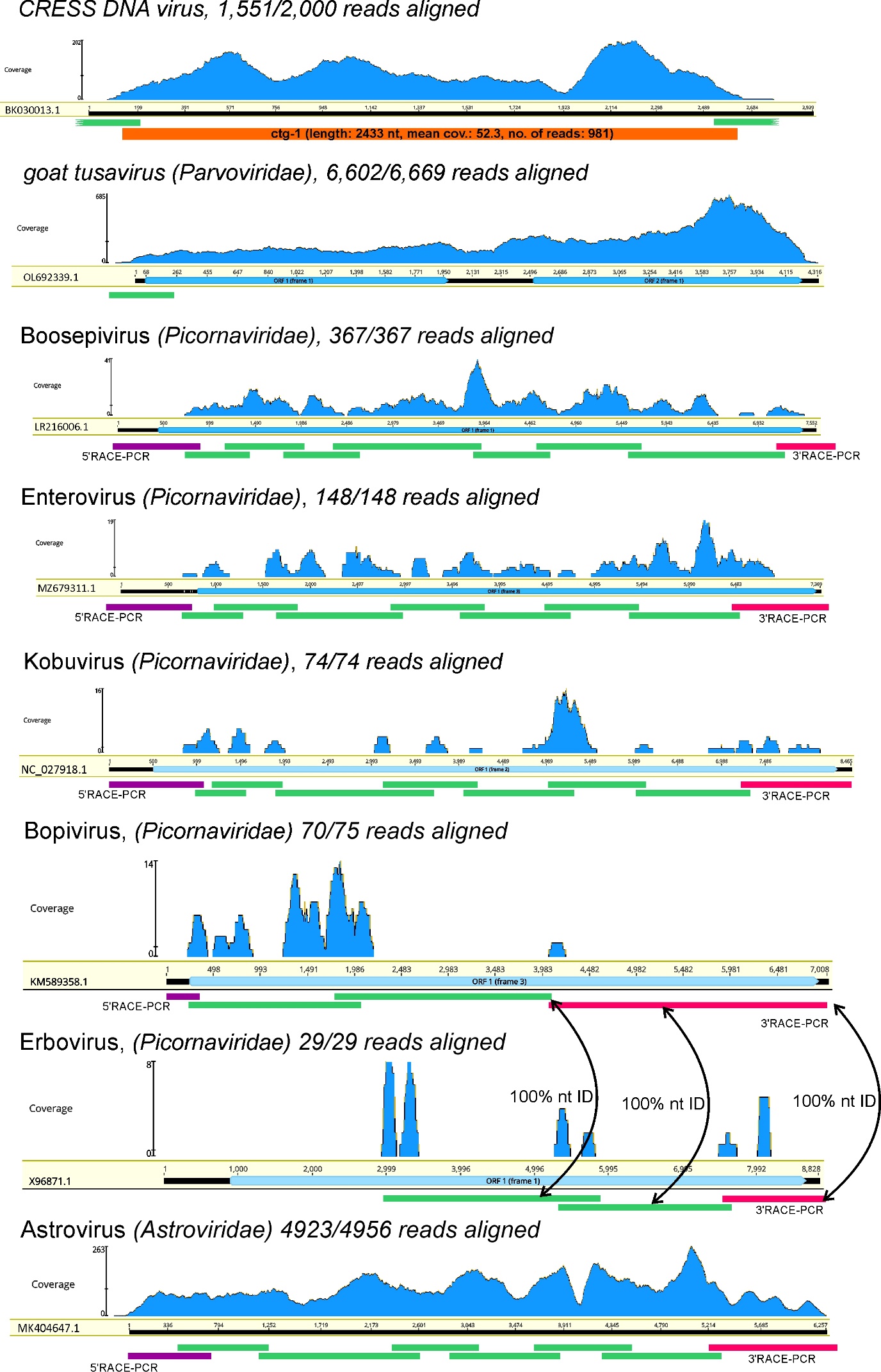


**Fig. S1**. Coverage maps (blue “mountains”) of the NGS reads mapped to selected reference genomes (yellow backgrounds) by Geneious Prime. Green, purple and magenta bars indicate the positions of a conventional (RT-)PCR, 5’ RACE and 3’ RACE PCR products used for complete genome/coding sequence characterization reactions, respectively. Double arrows indicate the localizations of PCR products with complete nt identities (ID). An orange bar shows the localization (and features, where mean cov. means mean coverage, no. of reads means number of reads used for the generation of the contig) of the longest contig (ctg-1) generated from the *de novo* assembly of the circovirus-reads by Geneious Prime.


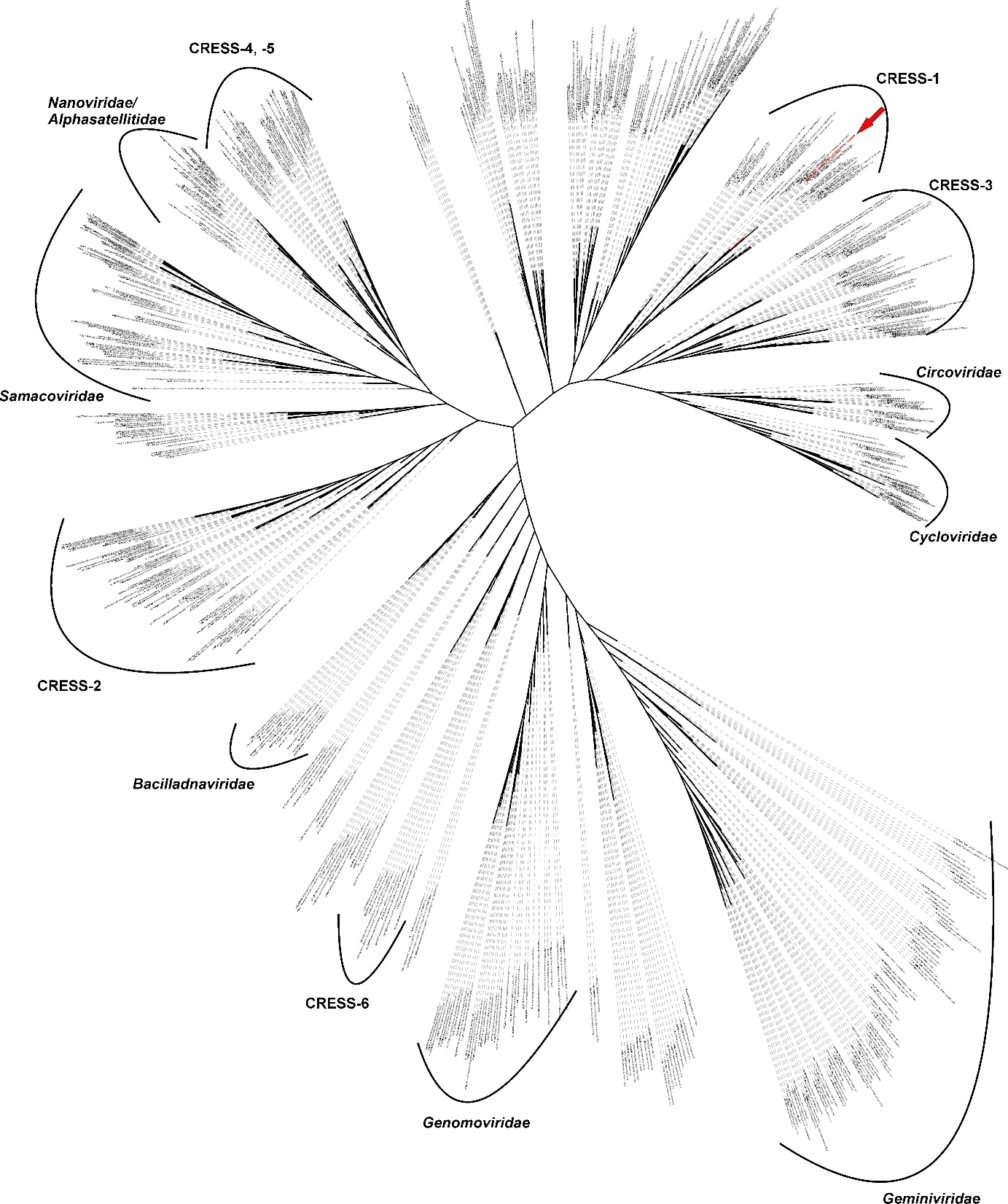


**Fig. S2**. Maximum-likelihood phylogenetic tree generated from a protein alignment of the full-length Rep proteins of goat/CV/KT-G4/2020-HUN (OQ758030, written in red and marked with a red arrow) and n=672 diverse CRESS DNA viruses of from the study of Kazlauskas and co-workers (6). The alignment was supplemented with additional Rep sequences that showed the highest sequence similarities to the study strain from the results of BLASTp searches. The phylogenetic positions of the official (with *italics*) and proposed (CRESS-1-6) CRESS DNA virus families are also indicated.


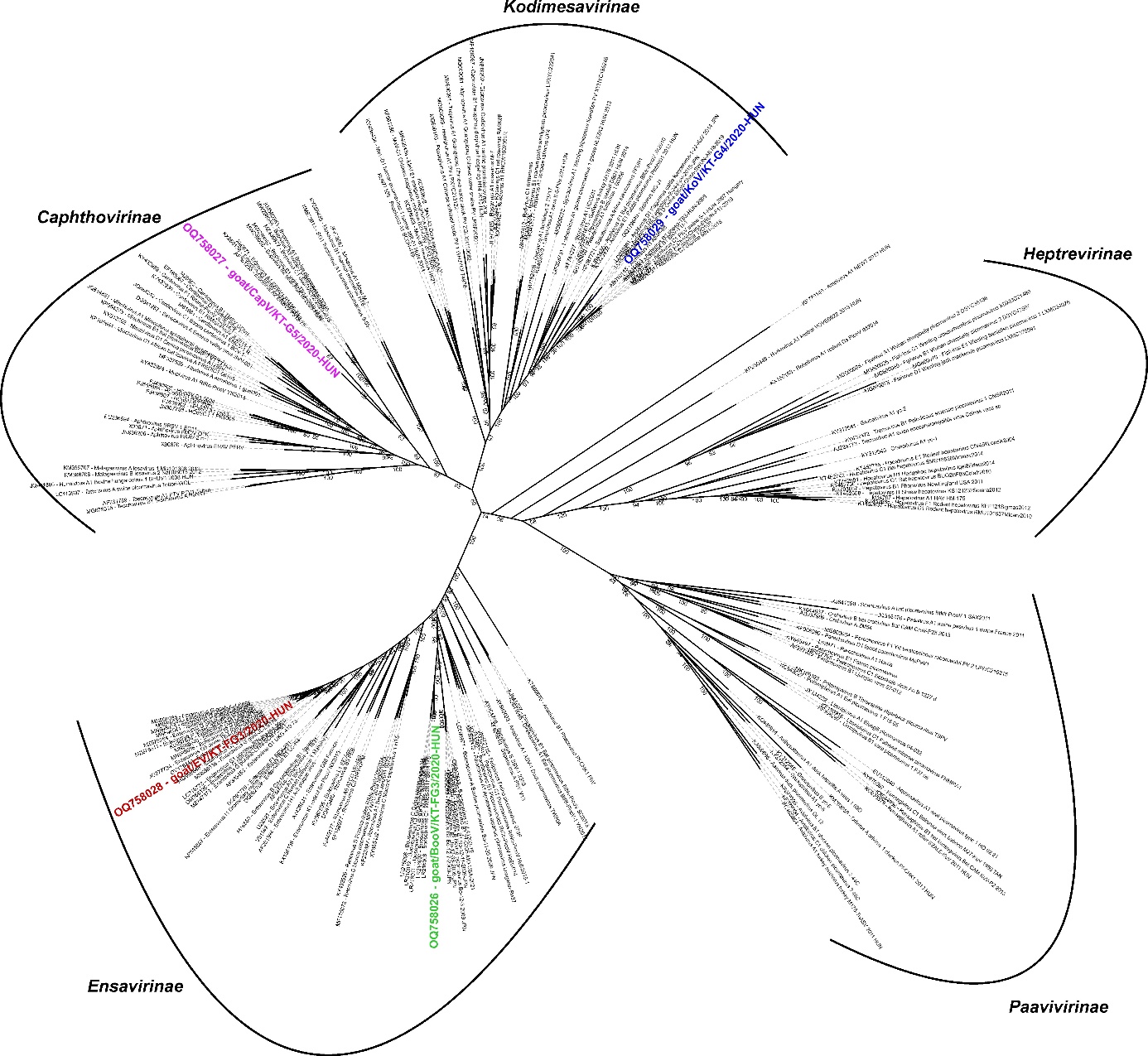


**Fig. S3.**: Phylogenetic analysis of the 3D nucleotide sequences of the study caprine picornaviruses (indicated with bold and distinct colours) and most similar sequences identified by BLAST searches together with other representative members of the *Picornaviridae* family. To help with orientation the phylogenetic positions of the five subfamilies are marked with arches. The tree was generated from the codon-based nt alignment of n=188 picornavirus sequences using IQtree with Ultrafast bootstrap (n=1000) and GTR+F+I+G4 model which was chosen automatically based on the BIC score.


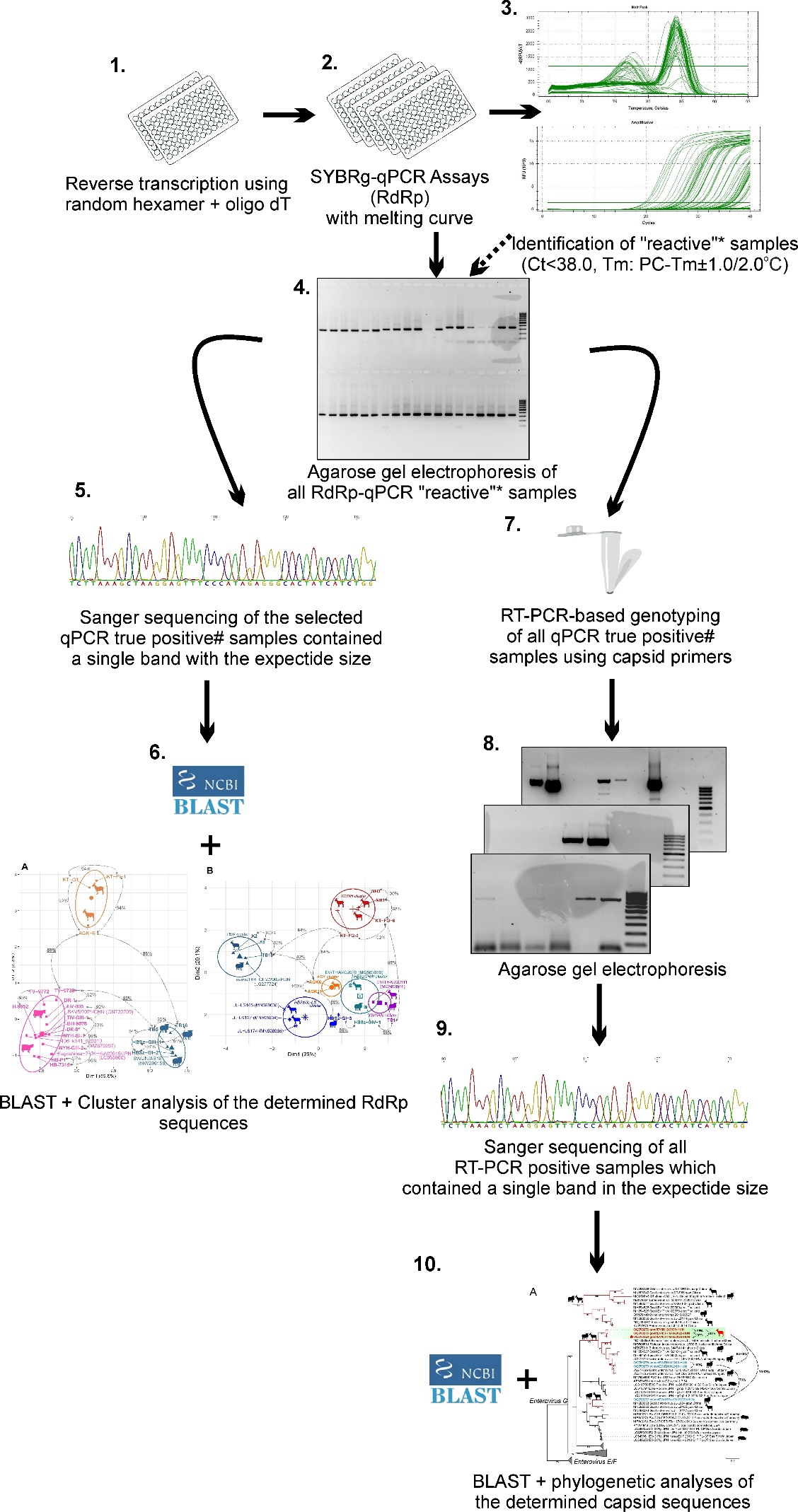


**Fig. S4:** Schematic representation of the step-by-step pipeline used for epidemiological investigations and genotyping reactions of selected virus groups using SYBRg-qPCR assays, classical RT-PCR reactions and dye terminator/Sanger sequencing.
